# Phylogenetic relationships within the Phyllidiidae (Opisthobranchia, Nudibranchia)

**DOI:** 10.3897/zookeys.605.7136

**Published:** 2016-07-14

**Authors:** Bart E.M.W. Stoffels, Sancia E.T. van der Meij, Bert W. Hoeksema, Joris van Alphen, Theo van Alen, Maria Angelica Meyers-Muñoz, Nicole J. de Voogd, Yosephine Tuti, Gerard van der Velde

**Affiliations:** 1 Radboud University Nijmegen, Institute for Water and Wetland Research, Heyendaalseweg 135, 6525 AJ Nijmegen, The Netherlands; 2 Naturalis Biodiversity Center, PO Box 9517, 2300 RA Leiden, The Netherlands; 3 Oxford University Museum of Natural History, Parks Road, Oxford OX1 3PW, United Kingdom; 4 Research Center for Oceanography (RCO), Indonesian Institute of Science (LIPI), Jl. Pasir Putih I, Ancol Timur, Jakarta 14430, Indonesia

**Keywords:** COI, Indonesia, mtDNA, nudibranch, phylogenetic relations, 16S

## Abstract

The Phyllidiidae (Gastropoda, Heterobranchia, Nudibranchia) is a family of colourful nudibranchs found on Indo-Pacific coral reefs. Despite the abundant and widespread occurrence of many species, their phylogenetic relationships are not well known. The present study is the first contribution to fill the gap in our knowledge on their phylogeny by combining morphological and molecular data. For that purpose 99 specimens belonging to 16 species were collected at two localities in Indonesia. They were photographed and used to make a phylogeny reconstruction based on newly obtained cytochrome oxidase subunit (COI) sequences as well as sequence data from GenBank. All mitochondrial 16S sequence data available from GenBank were used in a separate phylogeny reconstruction to obtain information for species we did not collect. COI data allowed the distinction of the genera and species, whereas the 16S data gave a mixed result with respect to the genera *Phyllidia* and *Phyllidiella*. Specimens which could be ascribed to species level based on their external morphology and colour patterns showed low variation in COI sequences, but there were two exceptions: three specimens identified as Phyllidia cf. babai represent two to three different species, while *Phyllidiella pustulosa* showed highly supported subclades. The barcoding marker COI also confirms that the species boundaries in morphologically highly variable species such as *Phyllidia elegans*, *Phyllidia varicosa*, and *Phyllidiopsis krempfi*, are correct as presently understood. In the COI as well as the 16S cladogram *Phyllidiopsis cardinalis* was located separately from all other Phyllidiidae, whereas *Phyllidiopsis fissuratus* was positioned alone from the *Phyllidiella* species by COI data only. Future studies on phyllidiid systematics should continue to combine morphological information with DNA sequences to obtain a clearer insight in their phylogeny.

cytochrome oxidase subunit

## Introduction

Nudibranch gastropod molluscs have traditionally been classified with the Infraclass Opisthobranchia Milne Edwards, 1848, which consists of more than 6000 species (Yonow 2008). Although this taxon is not monophyletic and therefore is considered obsolete (Schrödl et al. 2011), taxonomic works still refer to “opisthobranchs” for practical reasons (e.g. Uribe et al. 2013) and Opisthobranchia is considered an “Informal Group” among the Heterobranchia (Wägele et al. 2014). These animals form, ecologically and morphologically, one of the most diverse groups of marine gastropods (Wägele et al. 2014). To avoid use of their misnomer, this well-known group of marine animals can also be referred to as sea slugs (Yonow 2015). Among these, the Nudibranchia Cuvier, 1817 form the largest order with an estimated number of more than 2000 species (Gosliner et al. 2008), although also estimates of nearly 3000 species are known (Vonnemann et al. 2005).

Much work has already been done to elucidate the phylogeny of the opisthobranchs by molecular analyses (e.g., Wollscheid and Wägele 1999, Grande et al. 2004a, 2004b, Vonnemann et al. 2005, Turner and Wilson 2008, Maeda et al. 2010, Pola and Gosliner 2010), but most of the phylogenetic relationships still remain unclear at family, genus, and species level, especially with regards to the nudibranchs. All nudibranch species and many other sea slugs are predators, which usually can be observed together with their prey (Behrens 2005, Pola and Gosliner 2010, van Alphen et al. 2011). Only rarely they are found together with potential predators such as sea anemones, mushroom corals, and pycnogonids (Piel 1991, Behrens 2005, van der Meij and Reijnen 2012, Mehrotra et al. 2015).

The present study aims to clarify the phylogenetic relationships within the Phyllidiidae Rafinesque, 1814, belonging to the Doridacea (Bouchet and Rocroi 2005). This family consists of more than 100 species divided over five genera: *Ceratophyllidia* Eliot, 1903, *Phyllidia* Cuvier, 1797, *Phyllidiella* Bergh, 1869, *Phyllidiopsis* Bergh, 1875, and *Reticulidia* Brunckhorst, 1990 (Bouchet 2015). The genera *Fryeria* JE Gray, 1853, and *Reyfria* Yonow, 1986, have been synonymised with *Phyllidia* (Valdés and Gosliner 1999).

Most nudibranchs of the family Phyllidiidae are commonly encountered on coral reefs, where they can easily be noticed because of their aposomatic colouration, which serves to deter possible predators from eating them (Ritson-Williams and Paul 2007). Nevertheless, only eight phyllidiid COI sequences can be found in GenBank, as well as two 18S sequences and 17 16S sequences. There are only a few published studies that incorporate even a single member of Phyllidiidae into a phylogenetic tree (e.g. Wollscheid-Lengeling et al. 2001) and even fewer deal with phylogenetic relationships among Phyllidiidae. Among the latter, most are using anatomical characters (Brunckhorst 1993, Valdés and Gosliner 1999, Valdés 2001, 2002) and only two are known to include a molecular and phylogenetic analysis (Valdés 2003, Cheney et al. 2014).

Phyllidiid slugs are characterized by their oval elongate and tough bodies, which generally possess hard notal tubercles on the dorsal side. Although their colouration is a main character used for their identification, many species cannot be identified based on colouration alone owing to their high intra-specific colour variation. Structure and pattern of the notal tubercles are important characters for identification. Other distinctive features of the Phyllidiidae are the retractile lamellate rhinophores, the compact digestive gland mass, and the triaulic reproductive system (Brunckhorst 1993). Another important character diagnosing the Phyllidiidae is the possession of numerous subdermal calcareous spicules of different microstructures (Chang et al. 2013). The Phyllidiidae have no jaws or radula and lack the dorsal, circumanal circlet of gills that is typical of other dorids (Brunckhorst 1993).

To study the phylogenetic relationships within the Phyllidiidae, a molecular analysis was performed based on DNA sequence data of the mitochondrial cytochrome oxidase I (COI) gene, combined with external morphological assessments of material collected in two areas in eastern Indonesia, the Raja Ampat islands (West Papua) and Ternate, off western Halmahera (Moluccas). Both locations are situated in the centre of maximum marine biodiversity, also known as the Coral Triangle (Hoeksema 2007). In earlier studies, high numbers of phyllidiid species were recorded from this area: 13 from the Bismarck Sea, Papua New Guinea (Domínguez et al. 2007), eleven from Ambon (Moluccas, Indonesia) (Yonow 2011), and eleven from the South China Sea (Sachidhanandam et al. 2000). Therefore, both of our areas were expected to show a high number of phyllidiid species that could be used for the present study.

## Materials and methods

### Sampling

**Figure 1.**
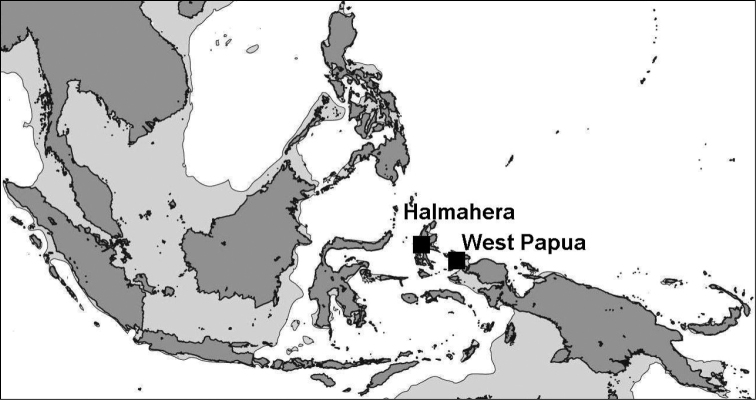
Location of field areas: Halmahera (including Ternate) and West Papua (including Raja Ampat).

**Figure 2.**
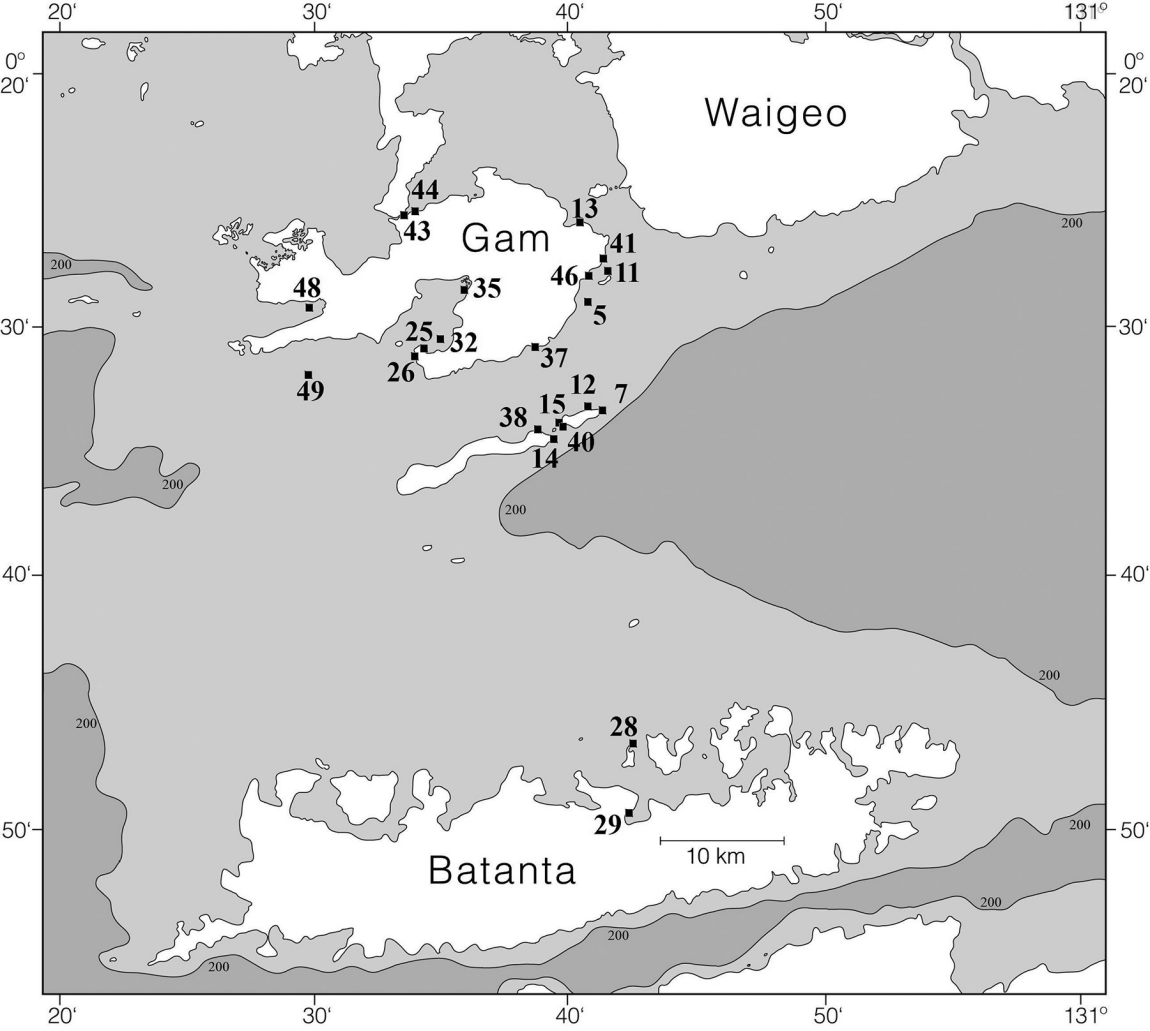
Raja Ampat sites where Phyllidiidae were sampled in 2007.

**Figure 3.**
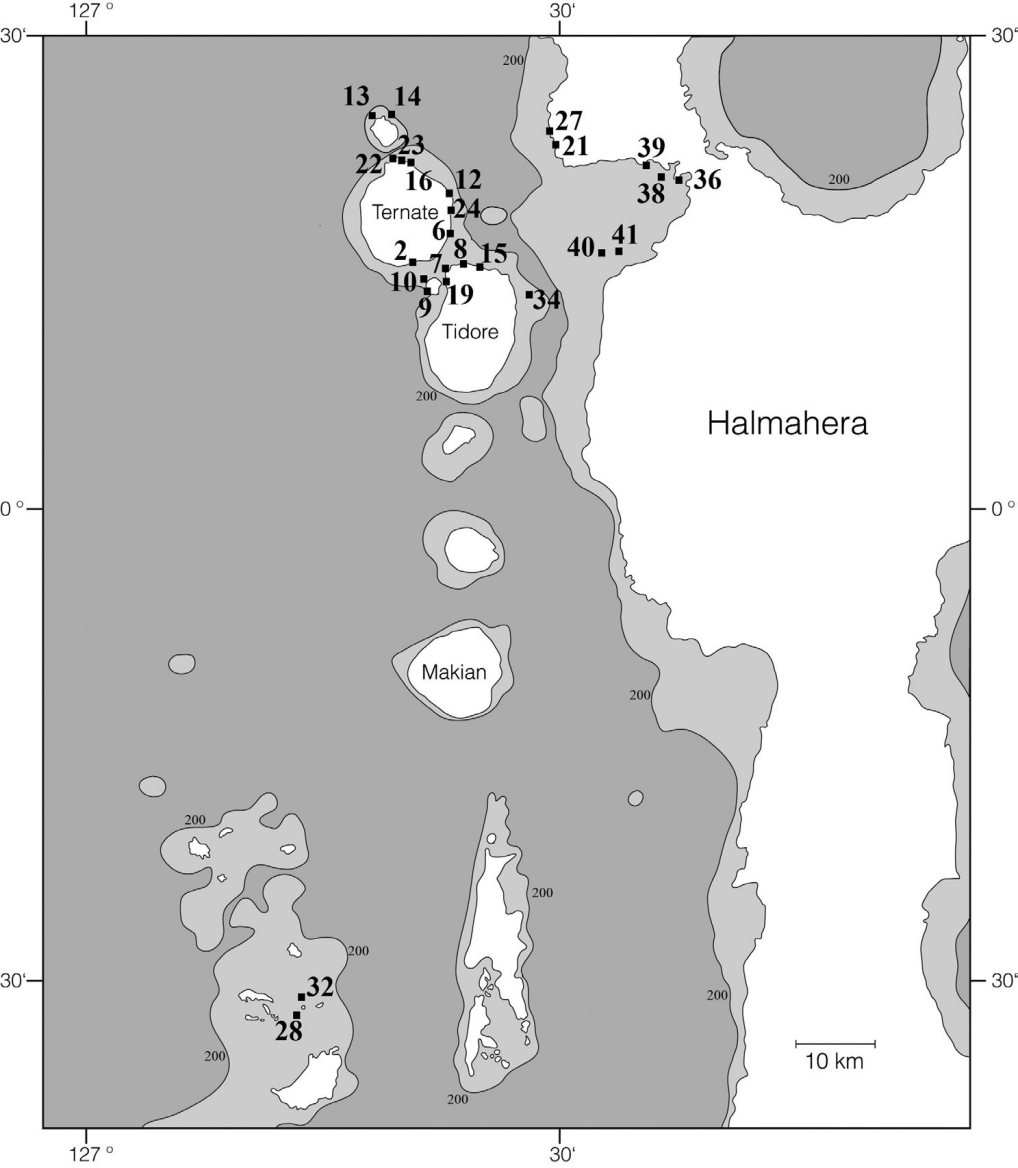
Halmahera and Ternate sites where Phyllidiidae were sampled in 2009.

Specimens were collected by SCUBA diving in West Papua by Gerard van der Velde in 2007, mostly in the coastal areas of Gam, Kri, Mansuar, and Batanta (Figures 1–2; see Hoeksema and van der Meij 2008). Additional specimens were mainly collected by Joris van Alphen and Nicole de Voogd, and also by Bert Hoeksema, Sancia van der Meij, and other expedition members (Hoeksema and van der Meij 2010) in 2009 off Halmahera (northern Moluccas), especially around Ternate (Figures 1, 3). A locality list of the sampling stations is provided in Table 1. Collected slugs were first photographed and subsequently preserved in 96% ethanol (West Papua 2007). Halmahera specimens were transferred into fresh 96% ethanol and labelled in order to prepare them for DNA analysis. These have been deposited in the mollusc collection of Naturalis Biodiversity Center, Leiden (coded as RMNH.Mol.), with the exception of some specimens that dried out after sequencing (Table 1; Figures 5–15; Suppl. material 1: COI sequences).

**Table 1.** Information on analysed Phyllidiidae species: RMNH.MOL catalogue number or field code number in case voucher specimen became lost; Genbank number if available; collection site, station number (RAJ = Raja Ampat, TER = Ternate, Halmahera), coordinates.

RMNH.MOL or Field nr.	Genbank accession number	Species	Locality	Station	Coordinates
336464	KX235918	*Phyllidia babai*	Tanjung Ebamadu	TER08	N0°45'23.4", E127°24'26.5"
336575	KX235920	Phyllidia cf. babai	South Gam, shoal near mangroves	RAJ37	S0°31'08.2", E130°38'28.0"
336614	KX235919	Phyllidia cf. babai	Tanjung Ratemu (South of river)	TER27	N0°54'44.5", E127°29'09.9"
336573	KX235921	*Phyllidia coelestis*	Eastern entrance of passage	RAJ44	S0°25'44.3", E130°33'56.8"
336574	KX235922	*Phyllidia coelestis*	Wallace Lake	RAJ13	S0°26'31.1", E130°41'08.0"
58		*Phyllidia elegans*	Pulau Maka	TER13	N0°54'42.7", E127°18'32.9"
137		*Phyllidia elegans*	Pulau Pilongga, North	TER34	N0°42'49.8", E127°28'45.4"
156		*Phyllidia elegans*	Teluk Dodinga; Karang Ngeli West	TER40	N0°46'25.3", E127°32'22.0"
336475	KX073972	*Phyllidia elegans*	Tanjung Tabam	TER12	N0°50'05.1", E127°23'10.0"
336478	KX073973	*Phyllidia elegans*	Pulau Maka	TER13	N0°54'42.7", E127°18'32.9"
336488	KX073974	*Phyllidia elegans*	Tanjung Pasir Putih	TER16	N0°51'50.4", E127°20'36.7"
336514	KX073975	*Phyllidia elegans*	Dufadufa / Benteng Toloko	TER24	N0°48'49.1", E127°23'21.6"
336515	KX073976	*Phyllidia elegans*	Idem	TER24	N0°48'49.1", E127°23'21.6"
336554	KX073985	*Phyllidia elegans*	Passage	RAJ43	S0°25'45.2", E130°33'37.3"
336555	KX073990	*Phyllidia elegans*	Akber Reef	RAJ14	S0°34'15.2", E130°39'33.7"
336556	KX073988	*Phyllidia elegans*	Passage	RAJ43	S0°25'45.2", E130°33'37.3"
336557	KX073987	*Phyllidia elegans*	Idem	RAJ43	S0°25'45.2", E130°33'37.3"
336558	KX073984	*Phyllidia elegans*	Southwest Pulau Kri	RAJ40	S0°33'58.1", E130°39'46.2"
336559	KX073991	*Phyllidia elegans*	South Gam, shoal near mangroves	RAJ37	S0°31'08.2", E130°38'28.0"
336560	KX073983	*Phyllidia elegans*	Southwest Pulau Kri	RAJ40	S0°33'58.1", E130°39'46.2"
336561	KX073986	*Phyllidia elegans*	Passage	RAJ43	S0°25'45.2", E130°33'37.3"
336562	KX073989	*Phyllidia elegans*	Akber Reef	RAJ14	S0°34'15.2", E130°39'33.7"
336628	KX073977	*Phyllidia elegans*	Pulau Gura Ici, East	TER32	S0°01'17.3", E127°14'17.2"
336629	KX073978	*Phyllidia elegans*	Idem	TER32	S0°01'17.3", E127°14'17.2"
336631	KX073979	*Phyllidia elegans*	Pulau Pilongga, North	TER34	N0°42'49.8", E127°28'45.4"
336632	KX073980	*Phyllidia elegans*	Idem	TER34	N0°42'49.8", E127°28'45.4"
336633	KX073981	*Phyllidia elegans*	Idem	TER34	N0°42'49.8", E127°28'45.4"
336649	KX073982	*Phyllidia elegans*	Teluk Dodinga; Karang Ngeli West	TER40	N0°46'25.3", E127°32'22.0"
336484	KX235923	*Phyllidia exquisita*	Tanjung Ngafauda	TER14	N0°54'38.3", E127°29'20.7"
336494	KX235924	*Phyllidia ocellata*	Southwest of Tobala	TER19	N0°44'56.6", E127°23'13.5"
336563	KX235926	*Phyllidia ocellata*	Southeast Gam, Friwen Wonda	RAJ11	S0°28'29.9", E130°41'54.8"
336564	KX235925	*Phyllidia ocellata*	Idem	RAJ11	S0°28'29.9", E130°41'54.8"
336565	KX235927	*Phyllidia picta*	South Gam, Shoal near mangroves	RAJ37	S0°31'08.2", E130°38'28.0"
336566	KX235929	*Phyllidia picta*	Passage	RAJ43	S0°25'45.2", E130°33'37.3"
336567	KX235928	*Phyllidia picta*	North Batanta, West Telok Gegenlol	RAJ29	S0°49'42.5", E130°42'42.0"
336619	KX235930	*Phyllidia* sp.	Pulau Popaco, East	TER28	S0°01'51.9", E127°14'01.8"
74		*Phyllidia varicosa*	Tanjung Pasir Putih	TER16	N0°51'50.4", E127°20'36.7"
336489	KX235931	*Phyllidia varicosa*	Idem	TER16	N0°51'50.4", E127°20'36.7"
336568	KX235942	*Phyllidia varicosa*	Northeast Pulau Mansuar	RAJ38	S0°34'05.0", E130°38'31.5"
336569	KX235941	*Phyllidia varicosa*	Idem	RAJ38	S0°34'05.0", E130°38'31.5"
336570	KX235943	*Phyllidia varicosa*	North Batanta, West Telok Gegenlol	RAJ29	S0°49'42.5", E130°42'42.0"
336571	KX235938	*Phyllidia varicosa*	South Gam, Eastern entrance Besir Bay, Cape Besir	RAJ25	S0°30'51.5", E130°34'11.5"
336572	KX235940	*Phyllidia varicosa*	Idem	RAJ25	S0°30'51.5", E130°34'11.5"
336604	KX235932	*Phyllidia varicosa*	East side Ternate Harbour (outside)	TER25	N0°46'55.3", E127°23'19.9"
336609	KX235933	*Phyllidia varicosa*	Pasir Lamo (West side)	TER26	N0°53'20.5", E127°27'34.2"
336612	KX235934	*Phyllidia varicosa*	Idem	TER26	N0°53'20.5", E127°27'34.2"
336617	KX235935	*Phyllidia varicosa*	Tanjung Ratemu (South of river)	TER27	N0°54'44.5", E127°29'09.9"
336621	KX235936	*Phyllidia varicosa*	Pulau Popaco E	TER28	S0°01'51.9", E127°14'01.8"
336637	KX235937	*Phyllidia varicosa*	Teluk Dodinga East; North of Pulau Jere	TER36	N0°50'47.8", E127°37'48.7"
336647	KX235939	*Phyllidia varicosa*	Teluk Dodinga, Karang Galiasa Kecil West	TER39	N0°51'09.1", E127°35'19.5"
336590	KX235944	*Phyllidiopsis fissuratus*	Yenweres Bay	RAJ46	S0°29'13.0", E130°40'23.6"
336589	KX235945	*Phyllidiella rudmani*	Southeast Gam, Friwen Wonda	RAJ11	S0°28'29.9", E130°41'54.8"
336434	KX235946	*Phyllidiella nigra*	Off Danau Laguna	TER02	N0°45'29.7", E127°20'59.2"
336471	KX235947	*Phyllidiella nigra*	Maitara Northwest	TER10	N0°44'32.0", E127°21'50.9"
336472	KX235948	*Phyllidiella nigra*	Idem	TER10	N0°44'32.0", E127°21'50.9"
336501	KX235949	*Phyllidiella nigra*	Sulamadaha I	TER22	N0°52'03.6", E127°19'33.1"
336505	KX235950	*Phyllidiella nigra*	Sulamadaha II	TER23	N0°52'02.0", E127°19'45.8"
336576	KX235952	*Phyllidiella nigra*	South Gam, Eastern entrance Besir Bay, Pulau Bun	RAJ26	S0°30'59.3", E130°33'48.7"
336577	KX235951	*Phyllidiella nigra*	South Gam, Southeast Besir Bay	RAJ32	S0°30'45.2", E130°35'00.1"
75F		*Phyllidiella pustulosa*	North Batanta, West Telok Gegenlol	RAJ29	S0°49'42.5", E130°42'42.0"
336436	KX235953	*Phyllidiella pustulosa*	Off Danau Laguna	TER02	N0°45'29.7", E127°20'59.2"
336460	KX235954	*Phyllidiella pustulosa*	Desa Tahua	TER07	N0°45'09.1", E127°23'31.3"
336461	KX235955	*Phyllidiella pustulosa*	Idem	TER07	N0°45'09.1", E127°23'31.3"
336470	KX235956	*Phyllidiella pustulosa*	Northwest side of Maitara	TER10	N0°44'32.0", E127°21'50.9"
336474	KX235957	*Phyllidiella pustulosa*	Tanjung Tabam	TER12	N0°50'05.1", E127°23'10.0"
336495	KX235958	*Phyllidiella pustulosa*	Tanjung Ratemu (South of river)	TER21	N0°54'24.7", E127°29'17.7"
336508	KX235959	*Phyllidiella pustulosa*	Dufadufa / Benteng Toloko	TER24	N0°48'49.1", E127°23'21.6"
336510	KX235960	*Phyllidiella pustulosa*	Idem	TER24	N0°48'49.1", E127°23'21.6"
336578	KX235965	*Phyllidiella pustulosa*	South Gam, Southeast Besir Bay	RAJ32	S0°30'45.2", E130°35'00.1"
336579	KX235971	*Phyllidiella pustulosa*	South Gam, Besir Bay	RAJ35	S0°48'58.3", E130°59'16.6"
336580	KX235967	*Phyllidiella pustulosa*	Southwest Pulau Kri	RAJ40	S0°33'58.1", E130°39'46.2"
336581	KX235963	*Phyllidiella pustulosa*	South Gam, Besir Bay	RAJ35	S0°48'58.3", E130°59'16.6"
336582	KX235968	*Phyllidiella pustulosa*	Southwest Pulau Kri	RAJ40	S0°33'58.1", E130°39'46.2"
336583	KX235964	*Phyllidiella pustulosa*	South Gam, East entrance Besir Bay, Cape Besir	RAJ25	S0°30'51.5", E130°34'11.5"
336584	KX235961	*Phyllidiella pustulosa*	West Pulau Yeben Kecil	RAJ48	S0°29'20.6", E130°30'04.9"
336585	KX235969	*Phyllidiella pustulosa*	Southeast Gam, Desa Besir	RAJ41	S0°27'48.1", E130°41'14.6"
336586	KX235966	*Phyllidiella pustulosa*	Idem	RAJ41	S0°27'48.1", E130°41'14.6"
336587	KX235962	*Phyllidiella pustulosa*	South Gam, Eastern entrance Besir Bay, Cape Besir	RAJ25	S0°30'51.5", E130°34'11.5"
336588	KX235970	*Phyllidiella pustulosa*	West Pulau Yeben Kecil	RAJ48	S0°29'20.6", E130°30'04.9"
336453	KX235972	*Phyllidiopsis krempfi*	Kampung Cina / Tapak 2	TER06	N0°47'15.0", E127°23'25.0"
336462	KX235973	*Phyllidiopsis krempfi*	Tanjung Ebamadu	TER08	N0°45'23.4", E127°24'26.5"
336466	KX235974	*Phyllidiopsis krempfi*	Idem	TER08	N0°45'23.4", E127°24'26.5"
336469	KX235975	*Phyllidiopsis krempfi*	West Maitara	TER09	N0°43'47.6", E127°21'44.7"
336512	KX235976	*Phyllidiopsis krempfi*	Dufadufa / Benteng Toloko	TER24	N0°48'49.1", E127°23'21.6"
336594	KX235979	*Phyllidiopsis krempfi*	Southwest Pulau Kri, Kuburan	RAJ15	S0°33'42.8", E130°39'40.4"
336595	KX235984	*Phyllidiopsis krempfi*	Southwest Pulau Kri	RAJ40	S0°33'58.1", E130°39'46.2"
336596	KX235983	*Phyllidiopsis krempfi*	Northwest Pulau Mansuar, Lalosi reef	RAJ49	S0°32'53.5", E130°29'51.1"
336597	KX235978	*Phyllidiopsis krempfi*	Southwest Pulau Kri, Kuburan	RAJ15	S0°33'42.8", E130°39'40.4"
336598	KX235980	*Phyllidiopsis krempfi*	North Batanta, North Pulau Yarifi	RAJ28	S0°46'46.7", E130°42'42.7"
336599	KX235982	*Phyllidiopsis krempfi*	East Kri, Sorido Wall	RAJ12	S0°33'13.2", E130°41'16.9"
336600	KX235981	*Phyllidiopsis krempfi*	Northeast Mansuar	RAJ38	S0°34'05.0", E130°38'31.5"
336650	KX235977	*Phyllidiopsis krempfi*	Teluk Dodinga; West Karang Ngeli	TER40	N0°46'25.3", E127°32'22.0"
336451	KX235985	*Phyllidiopsis shireenae*	Kampung Cina / Tapak 2	TER06	N0°47'15.0", E127°23'25.0"
336652	KX235986	*Phyllidiopsis shireenae*	Teluk Dodinga; East Karang Luelue	TER41	N0°46'32.8", E127°33'43.4"
336591	KX235987	*Phyllidiopsis xishaensis*	Southeast Gam, Pulau Kerupiar, Mike’s Point	RAJ05	S0°30'57.1", E130°40'22.1"
336592	KX235988	*Phyllidiopsis xishaensis*	East Pulau Kri, Cape Kri	RAJ07	S0°33'22.2", E130°41'28.7"
336593	KX235989	*Phyllidiopsis xishaensis*	Eastern entrance of passage	RAJ44	S0°25'44.3", E130°33'56.8"
336640	KX235990	*Reticulidia fungia*	East Teluk Dodinga; North of Pulau Jere	TER36	N0°50'47.8", E127°37'48.7"
336455	KX235991	*Reticulidia halgerda*	Kampung Cina / Tapak 2	TER06	N0°47'15.0", E127°23'25.0"

### Morphological study

**Figure 4.**
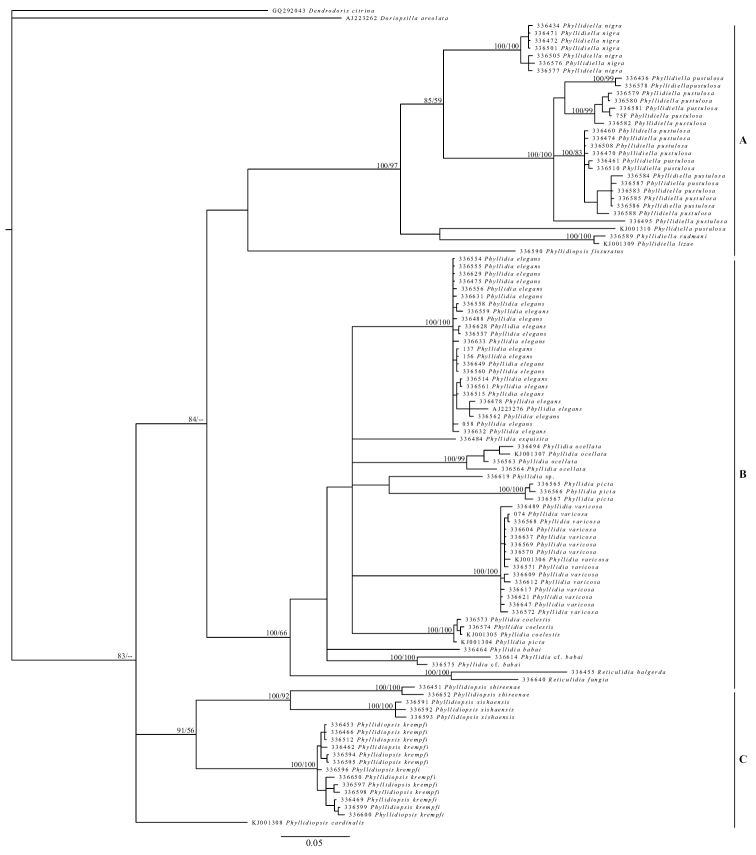
Phylogeny reconstruction of the Phyllidiidae based on COI gene sequence data of 109 specimens (including outgroups). Topology derived from Bayesian inference 50% majority rule, significance values are posterior probabilities / bootstrap values. Numbers refer to GenBank accession numbers / RMNH.Moll catalogue numbers.

**Figure 5.**
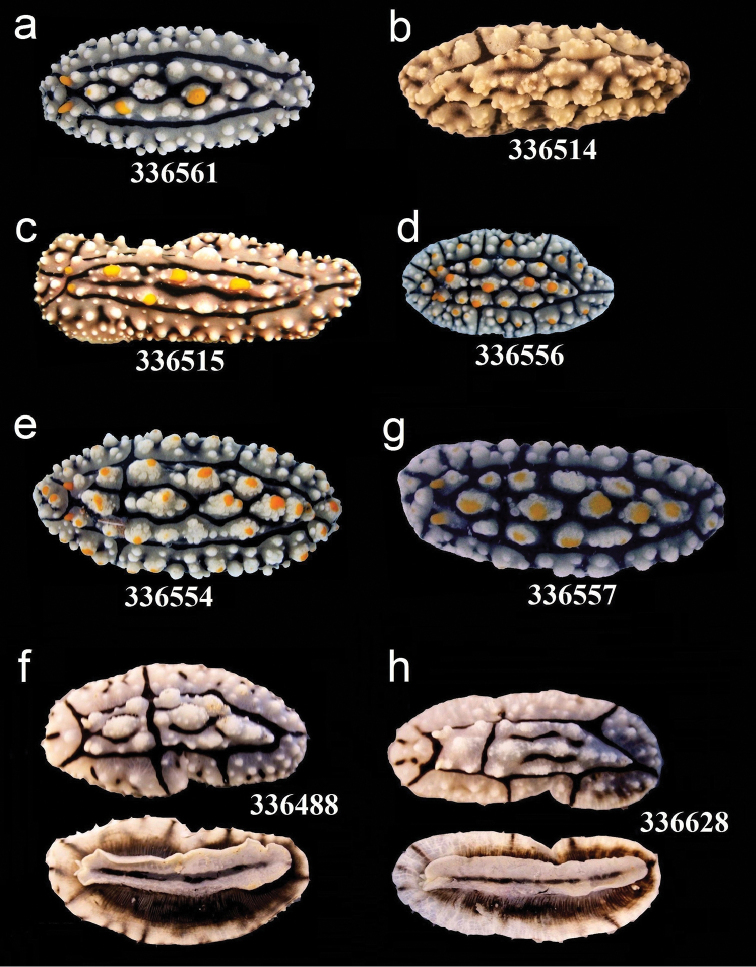
External morphology and colouration of Phyllidiidae specimens used for COI phylogeny reconstruction: *Phyllidia elegans*. Order of specimens (**a–h**) according to Figure 4 (**f**, **h** dorsal and ventral sides). Numbers refer to RMNH. Moll catalogue numbers.

**Figure 6.**
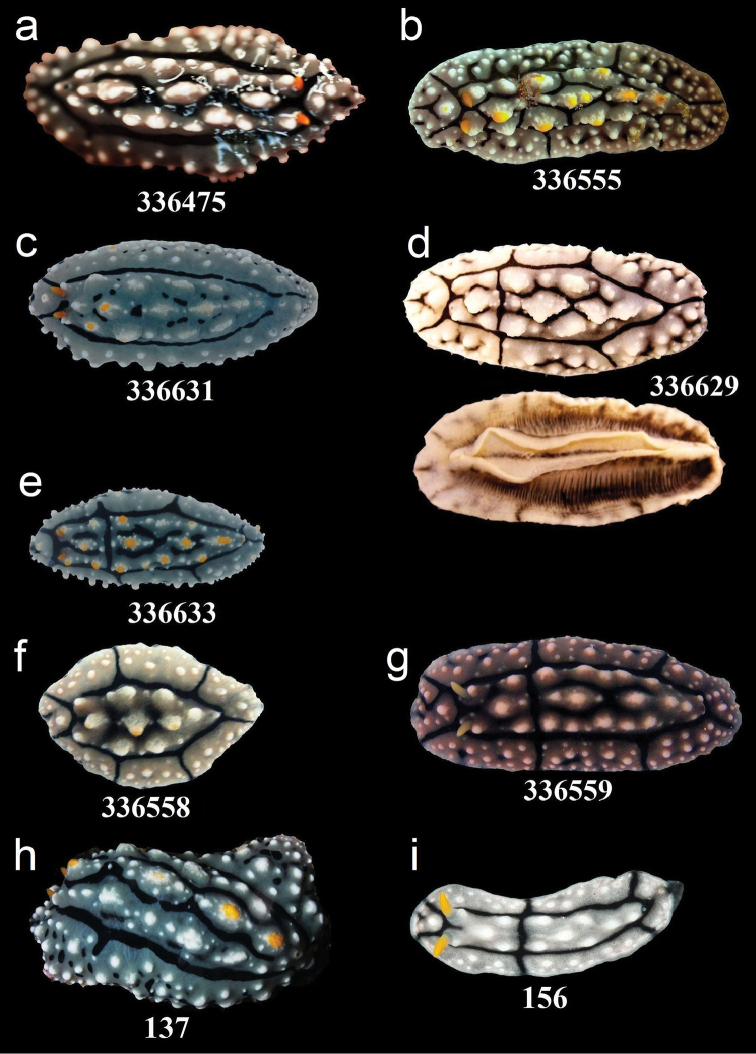
External morphology and colouration of Phyllidiidae specimens used for COI phylogeny reconstruction: *Phyllidia elegans*. Order of specimens (**a–i**) according to Figure 4 (**d** dorsal and ventral sides). Numbers refer to RMNH.Moll catalogue numbers and locality codes (137 and 156, dried-out).

**Figure 7.**
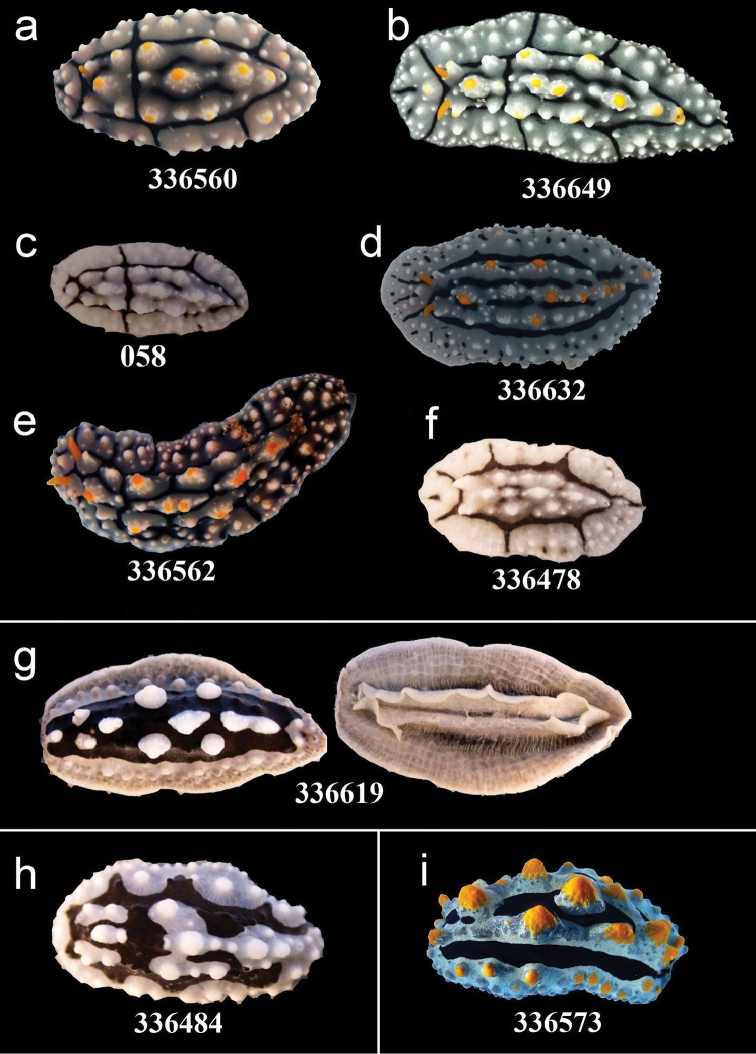
External morphology and colouration of Phyllidiidae specimens used for COI phylogeny reconstruction: *Phyllidia elegans* (**a–f**), *Phyllidia* sp. (**g** dorsal and ventral sides), *Phyllidia exquisita* (**h**), *Phyllidia coelestis* (**i**). Order of specimens (**a–i**) according to Figure 4. Numbers refer to RMNH.Moll catalogue numbers or locality code (058, dried-out).

**Figure 8.**
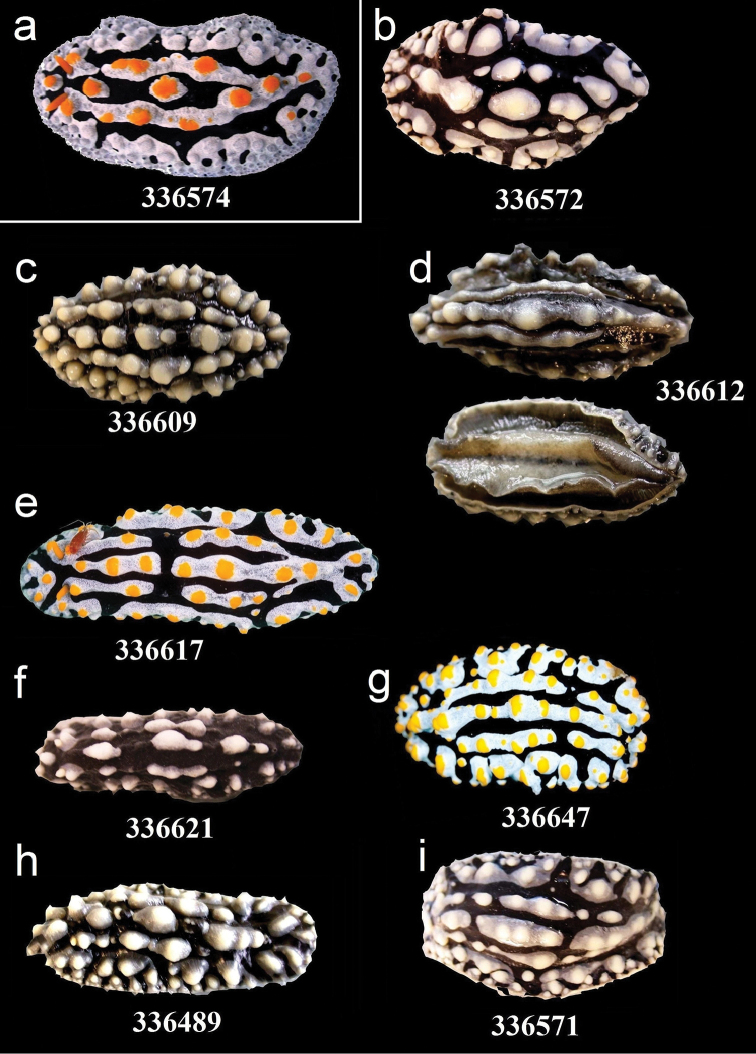
External morphology and colouration of Phyllidiidae specimens used for COI phylogeny reconstruction: *Phyllidia coelestis* (**a**), *Phyllidia varicosa* (**b–i**). Order of specimens (**a–i**) according to Figure 4 (**d** dorsal and ventral sides). Numbers refer to RMNH.Moll catalogue numbers.

**Figure 9.**
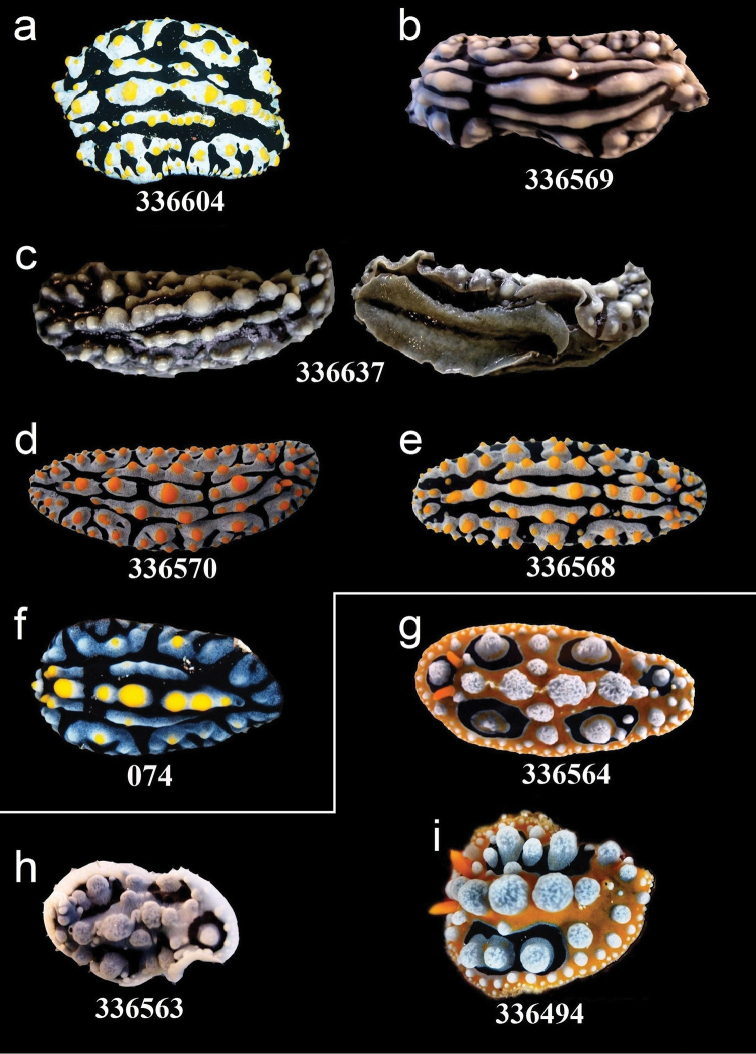
External morphology and colouration of Phyllidiidae specimens used for COI phylogeny reconstruction: *Phyllidia varicosa* (**a–f**), *Phyllidia ocellata* (**g–i**). Order of specimens (**a–i**) according to Figure 4 (**c** dorsal and ventral sides). Numbers refer to RMNH.Moll catalogue numbers or locality code (074, dried-out).

**Figure 10.**
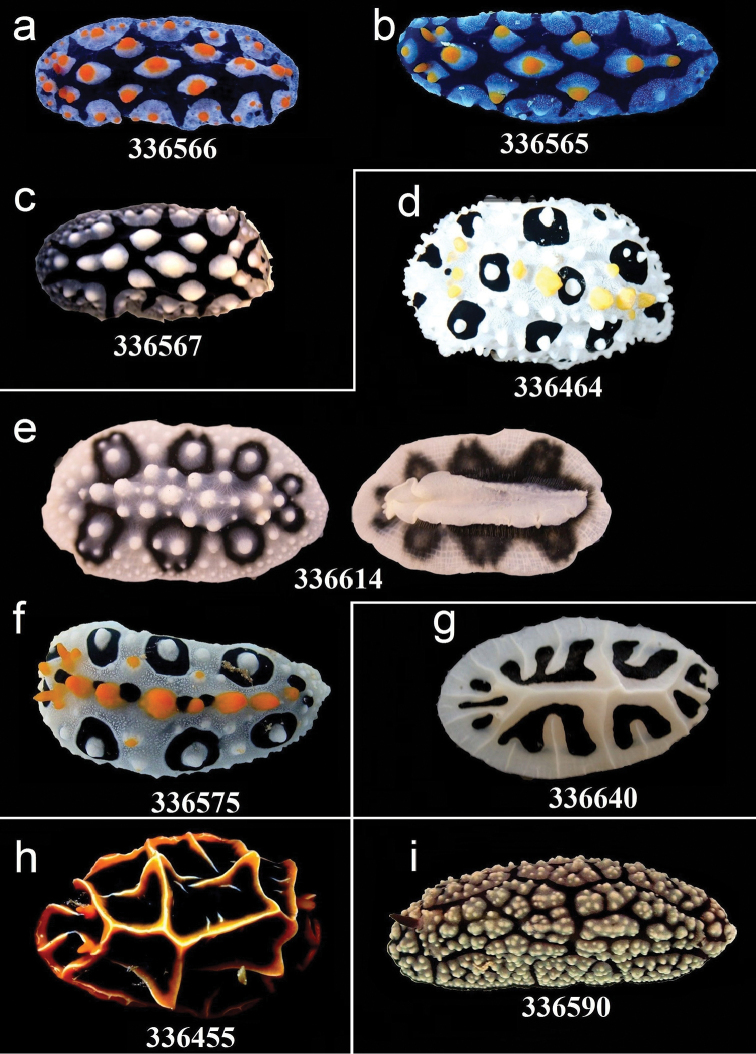
External morphology and colouration of Phyllidiidae specimens used for COI phylogeny reconstruction: *Phyllidia picta* (**a–c**), *Phyllidia babai* (**d**), Phyllidia cf. babai (**e–f**), *Reticulidia fungia* (**g**), *Reticulidia halgerda* (**h**), *Phyllidiopsis fissuratus* (**i**). Order of specimens (**a–i**) according to Figure 4 (**e** dorsal and ventral sides). Numbers refer to RMNH.Moll catalogue numbers.

**Figure 11.**
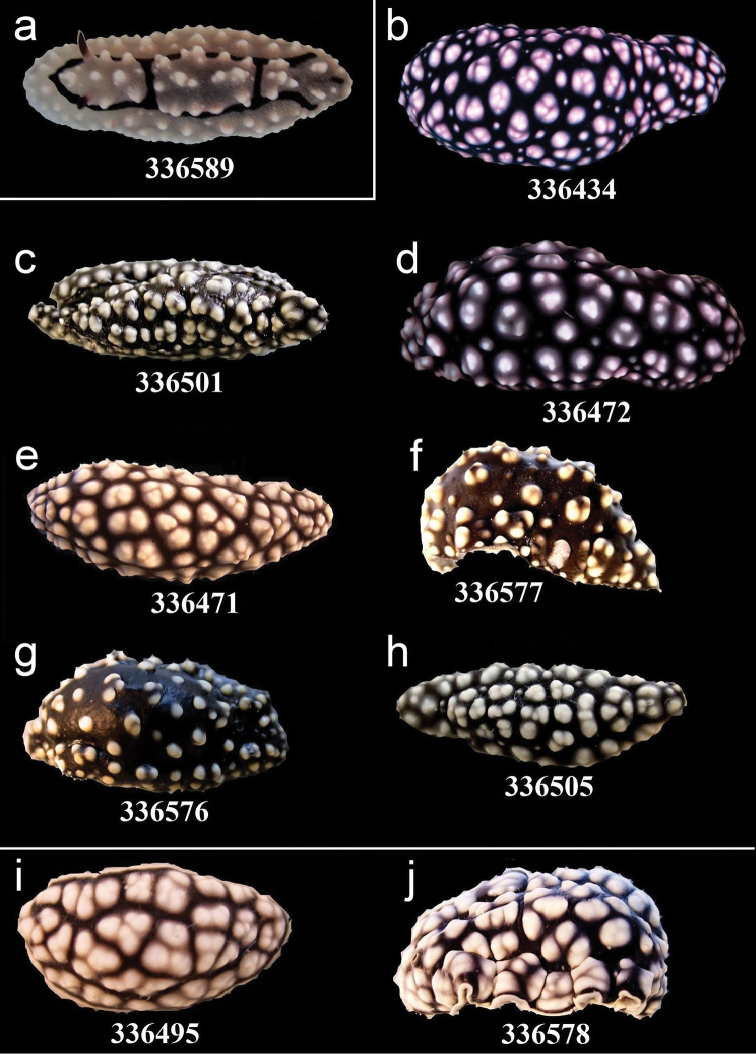
External morphology and colouration of Phyllidiidae specimens used for COI phylogeny reconstruction: *Phyllidiella rudmani* (**a**), *Phyllidiella nigra* (**b–h**), *Phyllidiella pustulosa* (**i–j**). Order of specimens (**a–j**) according to Figure 4. Numbers refer to RMNH.Moll catalogue numbers.

**Figure 12.**
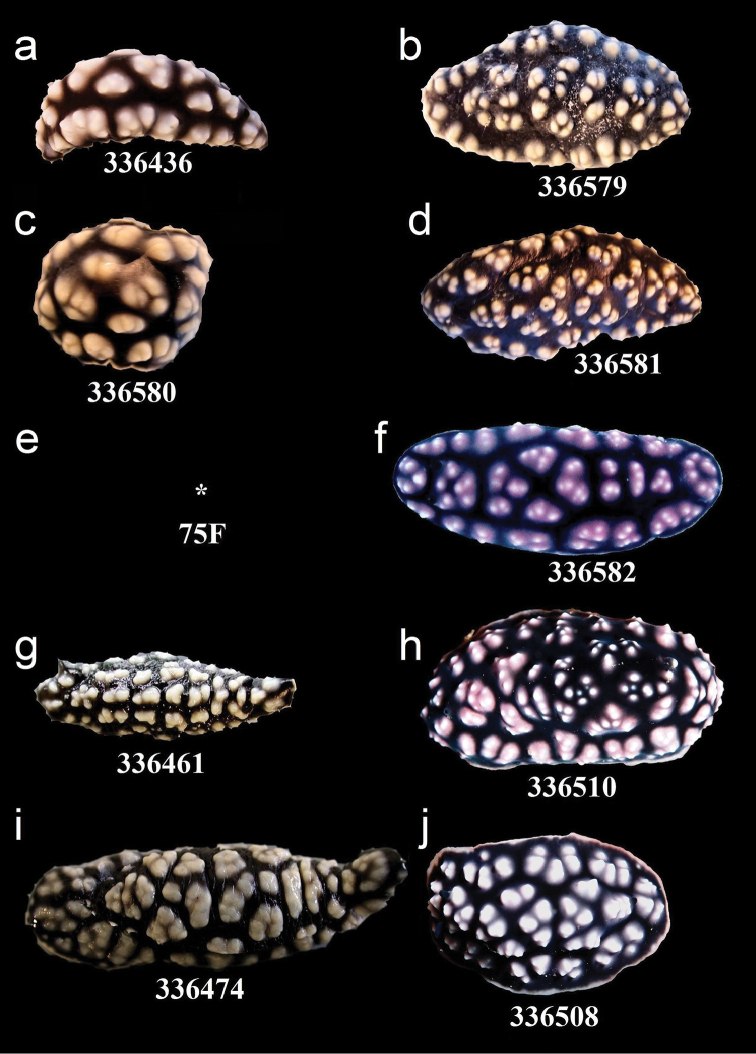
External morphology and colouration of Phyllidiidae specimens used for COI phylogeny reconstruction: *Phyllidiella pustulosa*. Order of specimens (**a–j**) according to Figure 4. Numbers refer to RMNH.Moll catalogue numbers or locality code (75F, dried-out).

**Figure 13.**
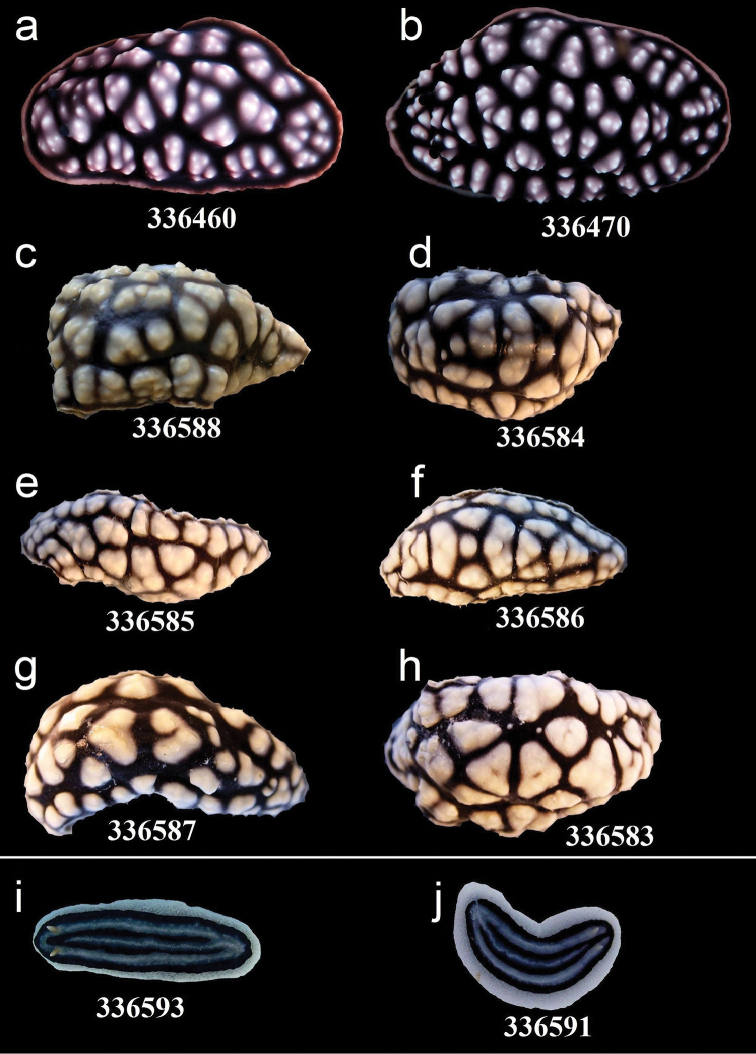
External morphology and colouration of Phyllidiidae specimens used for COI phylogeny reconstruction: *Phyllidiella pustulosa* (**a–h**), *Phyllidiopsis xishaensis* (**i–j**). Order of specimens (**a–j**) according to Figure 4. Numbers refer to RMNH.Moll catalogue numbers.

**Figure 14.**
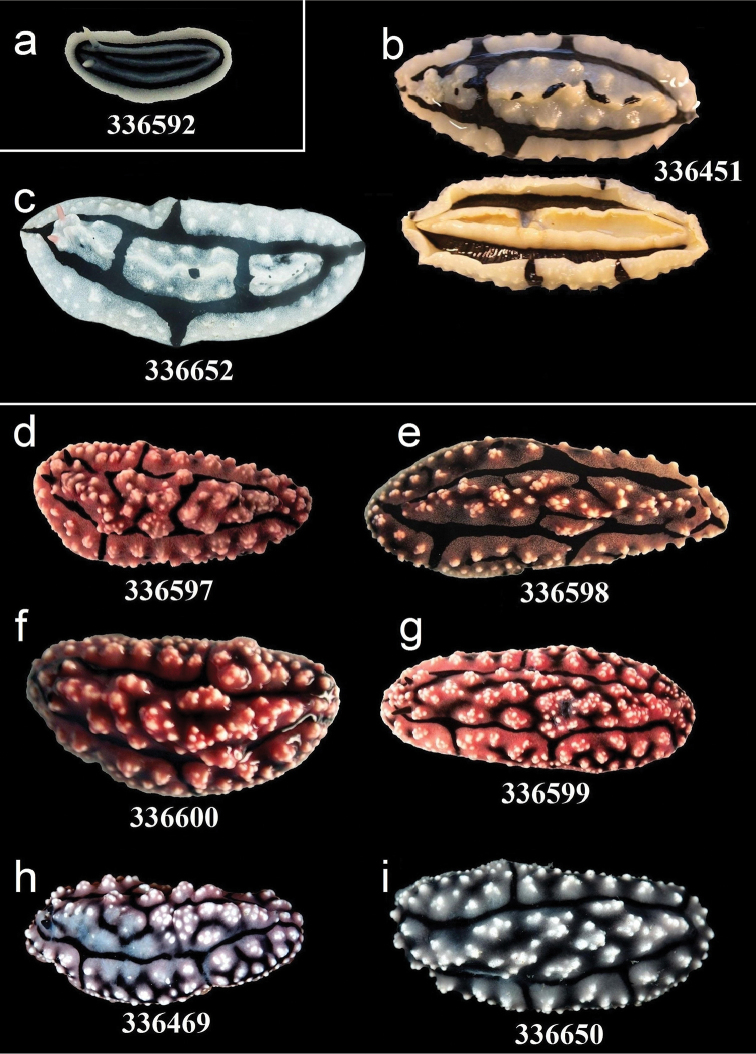
External morphology and colouration of Phyllidiidae specimens used for COI phylogeny reconstruction: *Phyllidiopsis xishaensis* (**a**), *Phyllidiopsis shireenae* (**b–c**), *Phyllidiopsis krempfi* (**d–i**). Order of specimens (**a–i**) according to Figure 4 (**c** dorsal and ventral sides). Numbers refer to RMNH.Moll catalogue numbers.

**Figure 15.**
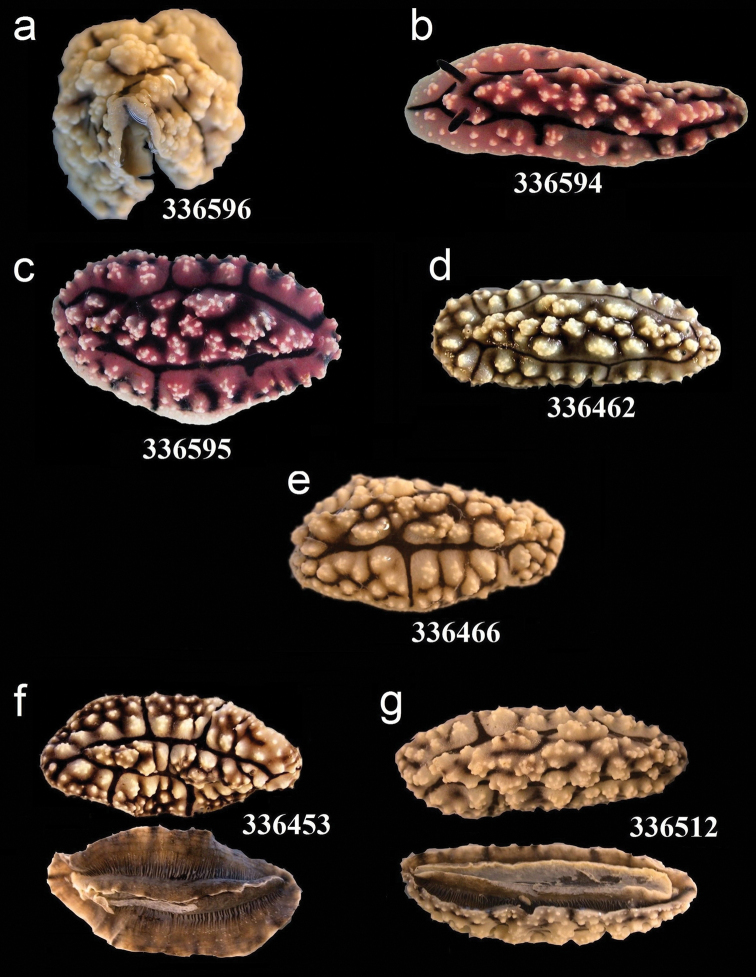
External morphology and colouration of Phyllidiidae specimens used for COI phylogeny reconstruction: *Phyllidiopsis krempfi*. Order of specimens (**a–g**) according to Figure 4 (**f**, **g** dorsal and ventral sides). Numbers refer to RMNH.Moll catalogue numbers.

Collected specimens were identified according to their external morphology using Brunckhorst (1993), Yonow et al. (2002), and Yonow (2011). In addition, field guides showing *in situ* photographs were used (Gosliner et al. 2008). All individuals except for three could be identified to species level. All specimens were photographed alive or in the preserved state (Figures 5–15); these photos can be linked to the phylogeny reconstruction of the Phyllidiidae based on COI gene sequence data (Figure 4).

### DNA extraction

For each species encountered in the field surveys one or more individuals were chosen for DNA analysis as well as from the morphologically distinct unidentified specimens, resulting in a total of 99 samples (Table 1). DNA was extracted from tissue of small foot fragments with the DNeasy Blood & Tissue Kit (Qiagen, Germany) according to the manufacturer’s protocol. DNA was eluted in DEPC treated water. The quality of the extracted DNA was tested by agarose gel (0.7%) electrophoresis.

### PCR amplification, purification, and sequencing

Extracted DNA was used for Polymerase Chain Reaction (PCR) to amplify fragments of the mitochondrial gene COI (cytochrome *c* oxidase subunit 1). The primers used for the amplification of the COI gene were: LCO1490 (5’GGT CAA CAA ATC ATA AAG ATA TTG G 3’) and HCO2198 (5’TAA ACT TCA GGG TGA CCA AAA AAT CA 3’) (Folmer et al. 1994). Thermal cycling conditions used for the amplification of the COI gene were: initial denaturing at 94 °C for 3 min followed by 38 amplification cycles of denaturation at 94 °C for 15 sec, primer annealing at 50 °C for 30 sec, and elongation at 72 °C for 1 min. A final elongation step at 72 °C for 5 min was performed. After checking by agarose (1%) electrophoresis if the PCR resulted the unique PCR fragments of the expected size (approximately 658 bp), the fragments were purified using the GeneJET PCR Purification Kit (Thermo Scientific, Landsmeer, NL). Purified PCR products were sequenced with corresponding primers.

### Sequence alignment and phylogenetic analyses

The quality of the sequences was checked using Chromas Lite (Technelysium Pty Ltd.). Subsequently the sequences were edited in MEGA 6 (Tamura et al. 2013) and analysed by BLAST searches (http://www.ncbi.nlm.nih.gov). COI sequences of *Dendrodoris citrina* (Cheeseman, 1881) and *Doriopsilla areolata* Bergh, 1880 were collected from GenBank and used as outgroups. Additional COI sequences of *Phyllidia coelestis* Bergh, 1905, *Phyllidia elegans* Bergh, 1869, *Phyllidia ocellata* Cuvier, 1804, *Phyllidia picta* Pruvot-Fol, 1957, *Phyllidia varicosa* Lamarck, 1801, *Phyllidiella lizae* Brunckhorst, 1993, *Phyllidiella pustulosa* (Cuvier, 1804), *Phyllidiopsis cardinalis* Bergh, 1875 were obtained from GenBank (Table 2).

**Table 2.** Mitochondrial COI sequences of Phyllidiidae (and outgroups) obtained from GenBank.

Species	Accession number	Reference	Collection locality
*Dendrodoris citrina*	GQ292043	Shields et al. (2009 unpubl.)	Ross Sea, Antarctica?
*Doriopsilla areolata*	AJ223262	Thollesson (2000)	Cadiz, Andalusia, Spain
*Phyllidia coelestis*	KJ001305	Cheney et al. (2014)	Lizard I., Queensland Australia
*Phyllidia elegans*	AJ223276	Thollesson (2000)	Tab I., Papua New Guinea
*Phyllidia ocellata*	KJ001307	Cheney et al. (2014)	Mooloolaba, Queensland, Australia
*Phyllidia picta*	KJ001304	Cheney et al. (2014)	Lizard I., Queensland Australia
*Phyllidia varicosa*	KJ001306	Cheney et al. (2014)	Lizard I., Queensland Australia
*Phyllidiella lizae*	KJ001309	Cheney et al. (2014)	Lizard I., Queensland Australia
*Phyllidiella pustulosa*	KJ001310	Cheney et al. (2014)	Lizard I., Queensland Australia
*Phyllidiopsis cardinalis*	KJ001308	Cheney et al. (2014)	Mooloolaba, Queensland, Australia

The newly obtained COI sequences and the sequences from GenBank were aligned using the Guidance server (Clustal W; Penn et al. 2010), resulting in an alignment score of 1.000. There were no unreliable columns. Prior to the model-based phylogenetic analysis, the best-fit model of nucleotide substitution was identified by means of the Akaike Information Criterion (AIC) calculated with jModeltest (Posada 2008), resulting in TVM+I+G as the most suitable model. Phylogenetic reconstructions were carried out with Bayesian inference in MrBayes 3.1.2 (Ronquist and Huelsenbeck 2003) using the most complex GTR+I+G model of nucleotide substitution. Bayesian inference coupled with Markov Chain Monte Carlo techniques (MCMC; six chains) were run for 5,000,000 generations with a sample tree saved every 1000 generations. The burnin was set to 25%. Likelihood scores stabilized at 0.007476. Consensus trees were visualized in FigTree v.1.3.1 (Rambaut 2009). A maximum likelihood analysis (GTR+I+G; 1000 bootstraps) was carried out with Phyml 3.1 (Guindon et al. 2010) using the Seaview platform (Gouy et al. 2010).

Initial phylogenetic analyses showed high intraspecific variation on the COI region between specimens identified as *Phyllidiella pustulosa*. Tests to estimate the average evolutionary divergence over sequence pairs between and within groups were carried out in MEGA 6.06. *Phyllidia elegans*, *Phyllidia varicosa*, *Phyllidiella nigra* (van Hasselt, 1824), *Phyllidiella pustulosa*, and *Phyllidiopsis krempfi* Pruvot-Fol, 1957 were used as representatives for each of the species groups, because of the larger number of available sequences for these species. The *Phyllidiella pustulosa* sequence from GenBank (KJ001310) was excluded from this analysis: based on its position in the phylogeny reconstruction the identification of this specimen as *Phyllidiella pustulosa* is doubtful. The web version of ABGD (Automatic Barcode Gap Discovery, Puillandre et al. 2012) was used to estimate the genetic distance corresponding to the difference between a speciation process versus intra-specific variation in *Phyllidiella pustulosa*. Runs were performed using the default range of priors (pmin = 0.001, pmax = 0.10) using the JC69 Jukes-Cantor measure of distance. The analysis involved 20 nucleotide sequences with a total of 588 positions in the final dataset.

All available mitochondrial 16S sequences of Phyllidiidae on GenBank (Tholesson 2000, Wolfscheid-Lengeling et al. 2001, Valdés 2003, Cheney et al. 2014, Shields et al. unpublished) were used for a phylogeny reconstruction based on this marker, which allowed us to study the phylogenetic position of 17 phyllidiid species including two species (*Phyllidia rueppelii* (Bergh, 1869) and *Phyllidiopsis sphingis* Brunckhorst, 1993) for which no COI data were available. *Doriopsilla albopunctata* (JG Cooper, 1863) was used as outgroup (Table 3). The sequences were aligned using the Guidance server (ClustalW; Penn et al. 2010), resulting in an alignment score of 0.996281. All unreliable columns (confidence score below 0.93) were removed. Prior to the model-based phylogenetic analysis, the best-fit model of nucleotide substitution was identified by means of the Akaike Information Criterion (AIC) calculated with jModeltest (Posada 2008), resulting in TVM+I+G. Because of the unavailability of TVM in MrBayes 3.1.2 (Ronquist and Huelsenbeck 2003), we used the most complex GTR+I+G model of nucleotide substitution. Bayesian inferences coupled with MCMC techniques (six chains) were run for 3,000,000 generations, with a sample tree saved every 1000 generations and the burnin set to 25%. Likelihood scores stabilized at a value of 0.005654. Consensus trees were visualized in FigTree v.1.3.1 (Rambaut 2009). A maximum likelihood analysis (GTR+I+G; 1000 bootstraps) was carried out with Phyml 3.1 (Guindon et al. 2010) using the Seaview platform (Gouy et al. 2010).

**Table 3.** 16S sequences of Phyllidiidae obtained from GenBank.

Species	Accession number	Reference	Collection locality
*Doropsilla albopunctata*	AF430354	Valdés (2003)	Baja California, Mexico
*Phyllidia coelestis*	AF430361	Valdés (2003)	Lifou I., New Caledonia
*Phyllidia coelestis*	KJ018917	Cheney et al. (2014)	Lizard I., Queensland Australia
*Phyllidia elegans*	AF430362	Valdés (2003)	Lifou I., New Caledonia
*Phyllidia elegans*	AJ225201	Thollesson (2000)	Tab I., Papua New Guinea
*Phyllidia ocellata*	AF430363	Valdés (2003)	Lifou I., New Caledonia
*Phyllidia picta*	KJ018916	Cheney et al. (2014)	Lizard I., Queensland Australia
*Phyllidia rueppelii*	AF430358	Valdés (2003)	Hurghada, Egypt
*Phyllidiella lizae*	AF430365	Valdés (2003)	Lifou I., New Caledonia
*Phyllidiella lizae*	KJ018918	Cheney et al. (2014)	Lizard I., Queensland Australia
*Phyllidiella pustulosa*	AF249232	Wollscheid-Lengeling et al. (2001)	Great Barrier Reef, Australia
*Phyllidiella pustulosa*	AF430366	Valdés (2003)	Lifou I., New Caledonia
*Phyllidia varicosa*	AF430364	Valdés (2003)	Lifou I., New Caledonia
*Phyllidiopsis cardinalis*	AF430367	Valdés (2003)	Lifou I., New Caledonia
*Phyllidiopsis sphingis*	AF430368	Valdés (2003)	Lifou I., New Caledonia
Phyllidiopsis xishaensis*	AF430369	Valdés (2003)	Lifou I., New Caledonia
*Reticulidia fungia*	AF430370	Valdés (2003)	Lifou I., New Caledonia
*Reticulidia halgerda*	AF430371	Valdés (2003)	Lifou I., New Caledonia

* Re-identification according to Yonow (pers. comm.)

## Results and discussion

### Position of genera

The reconstruction based on COI (Figure 4) is derived from the Bayesian inference 50% majority rule consensus. This topology is congruent with the one resulting from the maximum likelihood analysis. Three large groupings can be discerned (indicated as A, B, and C in Figure 4), albeit with low support for the higher taxonomic levels. The support values in the distal branches are high. The genera *Phyllidia*, *Phyllidiella*, *Phyllidiopsis*, and *Reticulidia* are retrieved in distinct clades, with *Reticulidia* as a sister clade to *Phyllidia*. *Phyllidiopsis fissuratus* Brunckhorst, 1993 formed a separate lineage basal to *Phyllidiella* species (albeit without support). *Phyllidiopsis cardinalis* does not cluster with its congeners, but instead forms a separate lineage in the Phyllidiidae.

The 16S phylogeny reconstruction is also derived from the Bayesian inference 50% majority rule consensus of the trees remaining after the burnin. There are low support values in the basal part of the tree and high support values in the distal phylogenetic branches (Figure 17). The Bayesian inference topology is congruent with the topology resulting from the maximum likelihood analysis. The outgroup *Doriopsilla albopunctata* is separated by a long branch. Within the overall clade four main groupings can be distinguished: *Phyllidiella*, *Phyllidiopsis*, and *Reticulidia*, and a mixed clade of *Phyllidiella* and *Phyllidia*. Based on this analysis only the genus *Reticulidia* is monophyletic. *Phyllidiopsis cardinalis* does not cluster with any of the other analysed taxa, and holds a separate position in the phylogeny reconstruction. The latter is in accordance with the COI reconstruction (Figure 4).

**Figure 16.**
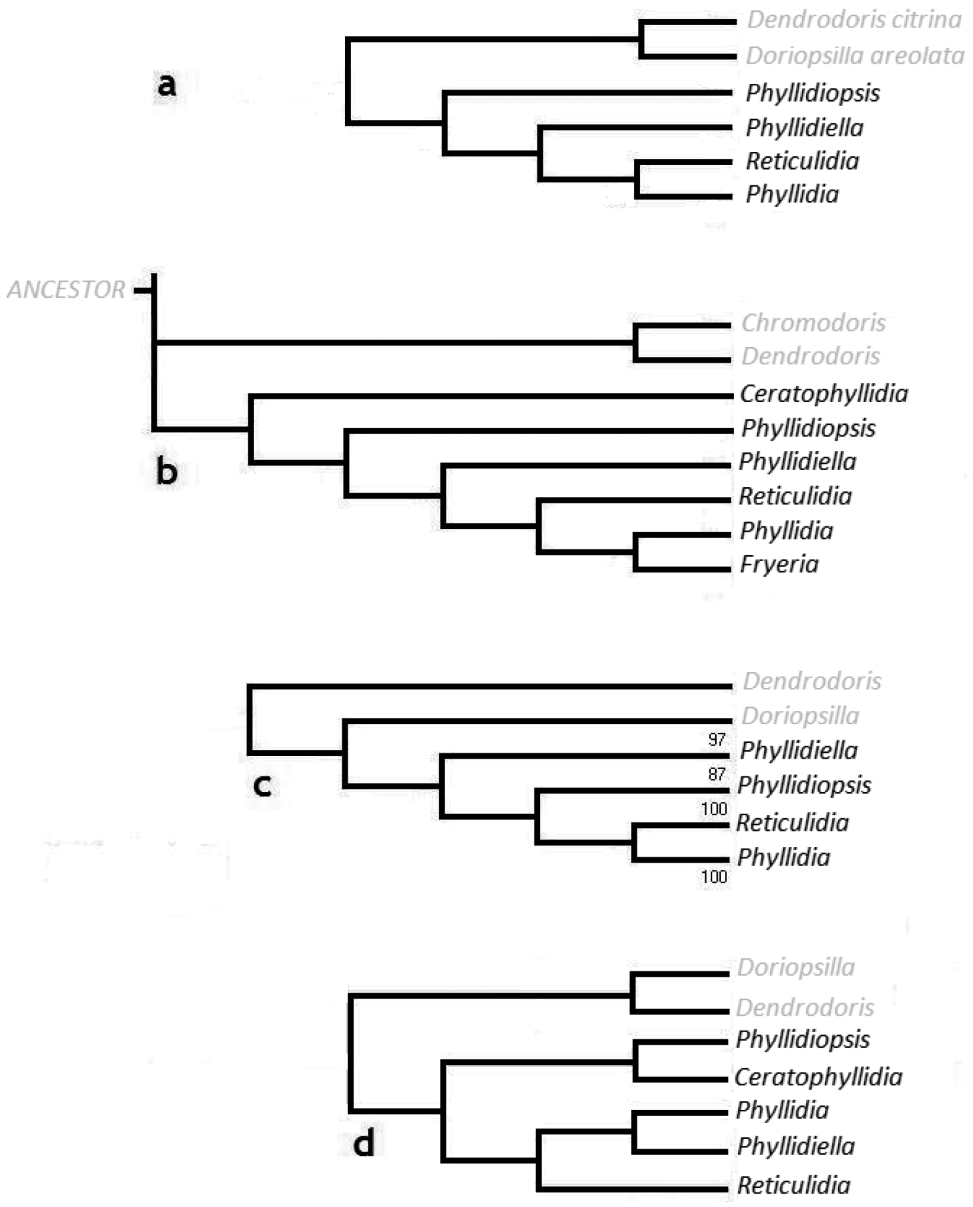
**a** Cladogram based on COI gene sequence data showing topology of four genera of Phyllidiidae **b** Cladogram according to Brunckhorst (1993) based on morphological data showing topology of six genera of Phyllidiidae **c** Cladogram based on 16S mtDNA sequence data showing topology of four genera of Phyllidiidae (Valdés 2003) **d** Cladogram based on morphological data (Valdés 2002) showing topology of five genera of Phyllidiidae.

The arrangement of the four phyllidiid genera based on the molecular data (Figures 4, 16a) is similar to that of Brunckhorst (1993) that was based on morphological and anatomical data (Figure 16b). The only exception is the position of the genus *Fryeria*. Brunckhorst (1993) distinguished *Fryeria* from *Phyllidia* based on the position of the anus and other anatomical features. *Phyllidia picta* (with its synonyms *Fryeria picta* (Pruvot-Fol, 1957), *Fryeria menindie* Brunckhorst, 1993, *Phyllidia menindie* (Brunckhorst, 1993)) was included in our analyses which, according to Brunckhorst, should belong to the genus *Fryeria*. Valdés and Gosliner (1999) synonymized both genera, which was later followed by Valdés (2003) and Cheney et al. (2014). The present reconstruction based on COI (Figure 16a) reconfirms the inclusion of *Fryeria* in the genus *Phyllidia*.

**Figure 17.**
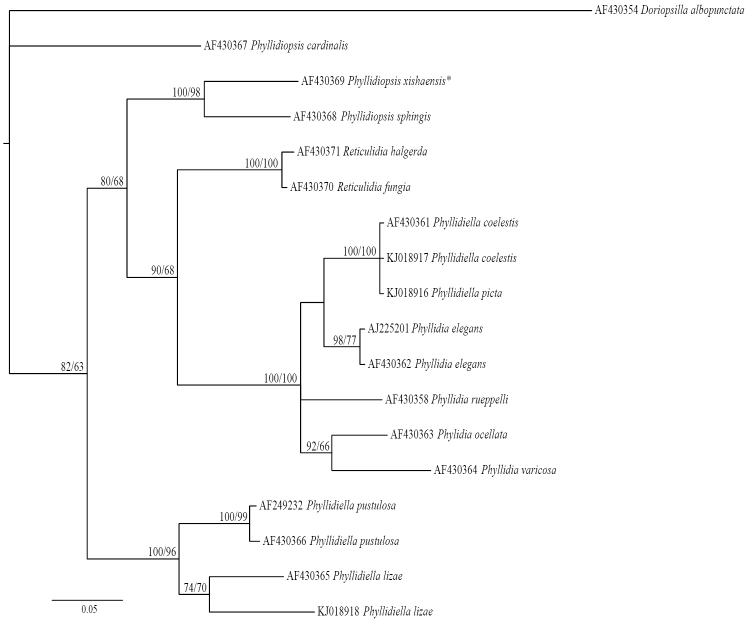
Phylogeny reconstruction of the Phyllidiidae based on 16S mtDNA of 17 specimens of 14 species (including outgroup). Topology derived from Bayesian inference 50% majority rule, significance values are posterior probabilities/bootstrap values. Numbers refer to GenBank accession numbers. *Re-identification according to Yonow (pers. comm.)

The cladogram of the genera based on 16S mtDNA sequence data collected by Valdés (2003) (Figure 16c) is roughly similar to the cladogram based on COI, except for the different positions of *Phyllidiopsis* and *Phyllidiella*. The cladogram based on morphological and anatomical data as shown by Valdés (2002; Figure 16d) is different from the other proposed classifications (Figures 16a–c). Brunckhorst (1993) considered *Ceratophyllidia* a sister group to all the other genera (Figure 6b). Valdés (2002; Figure 16d) distinguished two larger groupings within the Phyllidiidae; *Ceratophyllidia* and *Phyllidiopsis* as one group and *Phyllidia*, *Phyllidiella*, and *Reticulidia* as the other group. *Phyllidia* and *Phyllidiella* in turn formed a sister group of *Reticulidia* (Figure 16d). The cladogram by Brunckhorst (1993) and our cladogram based on COI (Figure 4) both show that *Phyllidiella* is a sister clade of *Reticulidia* and *Phyllidia*. In contrast, *Phyllidiella* is not a sister group of *Phyllidia* but to all the other genera grouped together in the cladogram of Valdés (2003).

Unfortunately no *Ceratophyllidia* specimens were available to complete our analysis at genus level. Up to this point the phylogenetic position of the genus *Ceratophyllidia* remains unclear, and additional molecular analyses are necessary to establish its position.

### Species level analysis

Species level analysis was mainly based on COI (Figure 4). Four nominal species were sequenced in the genus *Phyllidiella*. *Phyllidiella nigra* formed a highly supported clade. In the clade containing *Phyllidiella pustulosa* much variation is visible indicating larger genetic differences among individuals. The ABGD analysis shows that four Molecular Operational Taxonomic Units (MOTUs) are present in *Phyllidiella pustulosa*, suggesting the presence of cryptic species or, alternatively, high intraspecific variation. The *Phyllidiella pustulosa* of Cheney et al. (2014) falls in between the group consisting of *Phyllidiella nigra* and *Phyllidiella pustulosa* on one side and *Phyllidiella rudmani* Brunckhorst, 1993 on the other and probably represents another species. Our specimen of *Phyllidiella rudmani* clustered with the specimen identified as *Phyllidiella lizae* in Cheney et al. (2014). *Phyllidiella rudmani* and *Phyllidiella lizae* resemble each other (Brunckhorst 1993) and hence it is possible that the species identified as *Phyllidiella lizae* in Cheney et al. (2014) is in fact *Phyllidiella rudmani*.

Specimens of seven nominal *Phyllidia* species were sequenced. Sequences of 25 individuals of *Phyllidia elegans* (including one from GenBank) formed a highly supported clade, just like the clades containing *Phyllidia ocellata*, *Phyllidia picta*, and *Phyllidia varicosa. Phyllidia coelestis* was also retrieved as a highly supported clade. An individual identified as *Phyllidia picta* by Cheney et al. (2014) was part of this group suggesting that it should probably be identified as *Phyllidia coelestis*. Brunckhorst (1993) already noticed the close similarity between the two species but still confused them (Yonow 1996), and hence identification errors are likely to occur. Individuals identified as *Phyllidia babai* Brunckhorst, 1993 and Phyllidia cf. babai were retrieved in two different clades. Specimens 336464 and 336614 differ in 75 base pairs, 336464 and 336575 by 68 base pairs and 336614 and 336575 by 32 base pairs. Differences based on COI suggest that they represent two, or possibly three, different species. The genus *Reticulidia* was retrieved as a sister group of *Phyllidia*.

Material of four nominal species in the genus *Phyllidiopsis* was sequenced, with additional data of one species from GenBank (*Phyllidiopsis cardinalis*). *Phyllidiopsis fissuratus* clusters basal to *Phyllidiella*, without support. *Phyllidiopsis shireenae* Brunckhorst, 1990 and *Phyllidiopsis xishaensis* (Lin, 1983) cluster as sister species, in highly supported clades. *Phyllidiopsis krempfi* also formed a clear group. *Phyllidiopsis cardinalis* does not cluster with any of the phyllidiid genera based on either the 16S or the COI analysis. This result suggests that *Phyllidiopsis cardinalis* should be separated from the other *Phyllidiopsis* species, but further morphological analyses are needed to confirm this outcome. Brunckhorst (1993) noted that *Phyllidiopsis cardinalis* is the type species of the genus *Phyllidiopsis*, and that it has a unique and complex coloration totally different from that of any other known phyllidiid species, as well as a different anatomy, especially in the foregut. Valdés (2003) states “Additionally, the genus *Phyllidiopsis* is not monophyletic when molecular characters are used, because *Phyllidiopsis cardinalis* is at the base of the Phyllidiidae clade, and not nested with the other members of *Phyllidiopsis*”. Surprisingly, in the analysis of Cheney et al. (2014), based on a concatenated dataset of 16S and COI mtDNA, *Phyllidiopsis cardinalis* was retrieved in a highly supported clade with several species of *Phyllidiella* and *Phyllidia*.

### Variation within *Phyllidiella pustulosa*

*Phyllidiella pustulosa* is the only species in the COI cladogram (Figure 4) in which highly supported subclades can be discerned. To estimate the average evolutionary divergence within *Phyllidiella pustulosa* the base differences were compared per site for all grouped sequences of the species *Phyllidia elegans* (n = 24), *Phyllidia varicosa* (n = 15), *Phyllidiella nigra* (n = 7), *Phyllidiella pustulosa* (n = 20), and *Phyllidiopsis krempfi* (n = 13) (Tables 4–5).

**Table 4.** Estimates of average evolutionary divergence (p-distance) over sequence pairs within groups, in percentages.

Species	Distance (%)
*Phyllidia elegans*	0.7
*Phyllidia varicosa*	0.7
*Phyllidiella nigra*	0.6
*Phyllidiella pustulosa*	3.9
*Phyllidiopsis krempfi*	1.2

**Table 5.** Estimates of average evolutionary divergence (p-distance) over sequence pairs between groups, in percentages.

	Distance (%)				
Species	*Phyllidia elegans*	*Phyllidia varicosa*	*Phyllidiella nigra*	*Phyllidiella pustulosa*	*Phyllidiopsis krempfi*
*Phyllidia elegans*					
*Phyllidia varicosa*	12.1				
*Phyllidiella nigra*	15.8	15.5			
*Phyllidiella pustulosa*	18.3	18.9	10.5		
*Phyllidiopsis krempfi*	15.8	16.4	14.6	17.2	

The genetic variation on the barcoding marker COI is much higher within *Phyllidiella pustulosa* (3.9%) than within the other four species, which showed genetic variations between 0.6 and 1.2% (Table 4). The interspecific genetic variation (involving three different genera) ranges between 10.5 and 18.9% (Table 5). The congeners *Phyllidiella nigra* and *Phyllidiella pustulosa* differ by 10.5%, and the congeners *Phyllidia elegans* and *Phyllidia varicosa* differ by 12.1%. The observed levels of genetic variation within *Phyllidiella pustulosa* (Table 4) and between the five species (Table 5) call for additional studies on possible cryptic speciation in *Phyllidiella pustulosa*.

## Conclusions

The barcoding marker COI works well to separate the different species in the Phyllidiidae, and confirms that the species boundaries in highly variable species, such as *Phyllidia elegans*, *Phyllidia varicosa*, and *Phyllidiopsis krempfi*, are correct as presently understood. However, a multi-locus approach, preferably including nuclear markers, is needed to improve the resolution for the higher taxonomic levels. With the exception of a few species that are difficult to place (*Phyllidiopsis fissuratus*, *Phyllidiopsis cardinalis*) the studied genera (*Phyllidia*, *Phyllidiella*, *Phyllidiopsis*, and *Reticulidia*) were retrieved as separate genera within the family. Additional representatives of *Ceratophyllidia* are needed to indicate the position of this genus within the Phyllidiidae. The observed groupings within *Phyllidiella pustulosa* suggest that multiple (cryptic) species could be present in this species, for which further analyses are needed including morphological data and multiple markers. Chang and Willan (2015) indicated that at least nine clades could be recognized in *Phyllidiella pustulosa* that could be separated slightly according to morphological characters. We recommend that future studies combine DNA sequences with morphological characters, which can easily be done by adding pictures of the specimens to avoid increasing confusion in the identification of specimens.

## Supplementary materials

Supplementary material 1
COI sequences of lost Phyllidiidae specimens Data type: Adobe PDF file Explanation note: COI sequences of Phyllidiidae specimens that dried out after sequencing (numbers and localities are indicated in Table 1. This dataset is made available under the Open Database License (http://opendatacommons.org/licenses/odbl/1.0/). The Open Database License (ODbL) is a license agreement intended to allow users to freely share, modify, and use this Dataset while maintaining this same freedom for others, provided that the original source and author(s) are credited.Bart E.M.W. Stoffels, Sancia E.T. van der Meij, Bert W. Hoeksema, Joris van Alphen, Theo van Alen, Maria Angelica Meyers-Muñoz, Nicole J. de Voogd, Yosephine Tuti, Gerard van der Velde

## References

[B1] Behrens DW (2005) Nudibranch behavior. New World Publications, Jacksonville, 176 pp.

[B2] Bergh R (1869) Bidragtil en Monographi af Phillidierne. Naturhistorisk Tidsskrift, Kjobenhavn, Series 3, 5: 357–542. [pls. 14–24]

[B3] Bergh R (1875) Neue Beiträge zur Kenntniss der Phyllidiaden. Verhandlung der königlich-kaiserlich zoologisch-botanischen Gesellschaft in Wien (Abhandlungen) 25: 659–674. [pl. 16]

[B4] Bergh R (1880) Die Doriopsiden des Mittelmeeres. Jahrbücher Deutsche malakozoologische Gesellschaft 7: 297–328. [pls. 10–11]

[B5] Bergh R (1905) Die Opisthobranchiata der Siboga-Expedition (1899-1900). Siboga-Expeditie Monographie 50: 1–248. [pls. 1–20]

[B6] Bouchet P (2015) Phyllidiidae Rafinesque, 1814. World Register of Marine Species. http://www.marinespecies.org/aphia.php?p=taxdetails&id=23093 [on 2015-09-23]

[B7] Bouchet P Rocroi J-P (2005) Classification and nomenclature of gastropod families. Malacologia 47: 1–397.

[B8] Brunckhorst DJ (1990a) Description of a new genus and species, belonging to the family Phyllidiidae (Doridoidea). Journal of Molluscan Studies 56: 567–576. doi: 10.1093/mollus/56.4.567

[B9] Brunckhorst DJ (1990b) Description of a new species of *Phyllidiopsis* Bergh (Nudibranchia: Doridoidea: Phyllidiidae) from the tropical western Pacific, with comments on the Atlantic species. Journal of Molluscan Studies 56: 577–584. doi: 10.1093/mollus/56.4.577

[B10] Brunckhorst DJ (1993) The systematics and phylogeny of phyllidiid nudibranchs (Doridoidea). Records of the Australian Museum (Suppl) 16: 1–107. doi: 10.3853/j.0812-7387.16.1993.79

[B11] Chang Y-W Willan RC Mok H-K (2013) Can the morphology of the integumentary spicules be used to distinguish genera and species of phyllidiid nudibranchs (Porostomata: Phyllidiidae)? Molluscan Research 33: 14–23. doi: 10.1080/13235818.2012.754144

[B12] Chang Y-W Willan RC (2015) Molecular phylogeny of phyllidiid nudibranchs (Porostomata: Phyllidiidae) based on the mitochondrial genes (COI and 16S). Molluscs 2015 Program and Abstract Handbook, Triennial Conference of the Malacological Society of Australasia, 27.

[B13] Cheeseman TF (1881) On some new species of Nudibranchiate Mollusca. Transactions and Proceedings of the New Zealand Institute 8: 222–224.

[B14] Cheney KL Cortes F How MJ Wilson NG Blomberg SP Winters AE Umanzor S Marshall NJ (2014) Conspicuous visual signals do not coevolve with increased body size in marine slugs. Journal of Evolutionary Biology 27: 676–687. doi: 10.1111/jeb.12348 24588922 10.1111/jeb.12348

[B15] Cooper JG (1863) On new or rare Mollusca inhabiting the coast of California. No. II. Proceedings of the California Academy of Natural Sciences 3: 56–60.

[B16] Cuvier GLCF (1797) Sur un nouveau genre de mollusque. Bulletin des Sciences de la Société Philomathique de Paris 1(1): 105.

[B17] Cuvier GLCF (1804a) Mémoire sur la Phyllidie et sur le Pleurobranch, deux nouveaux genres de mollusques de l’ordre de gastéropodes, et voisin des patelles de les oscabrions, dont l’autre porte une coquille cachée. Annales du Muséum National d’Histoire Naturelles, Paris 5: 266–276.

[B18] Cuvier GLCF (1804b) Suite de mémoires sur les mollusques, par M. Cuvier, sur les genres Phyllidie et Pleurobranche. Bulletin des Sciences de la Société Philomathique de Paris 3(96): 277–278.

[B19] Cuvier GLCF (1817) Le règne animal distribué d’après son organisation, tome 2 contenant les reptiles, les poissons, les mollusques, les annélides. Deterville, Paris, 532 pp.

[B20] Domínguez M Quintas P Troncoso JS (2007) Phyllidiidae (Opisthobranchia: Nudibranchia) from Papua New Guinea with the description of a new species of *Phyllidiella*. American Malacological Bulletin 22: 89–117. doi: 10.4003/0740-2783-22.1.89

[B21] Eliot (1903) On some nudibranchs from East Africa and Zanzibar.2. Proceedings of the Zoological Society, London 1903: 250–253.

[B22] Folmer O Black M Hoeh W Lutz R Vrijenhoek R (1994) DNA primers for amplification of mitochondrial cytochrome c oxidase subunit I from diverse metazoan invertebrates. Molecular Marine Biology and Biotechnology 3: 294–299. doi: 10.1186/1472-6785-13-34 7881515

[B23] Gofas S (2015) Opisthobranchia. World Register of Marine Species (WoRMS). http://www.marinespecies.org/aphia.php?p=taxdetails&id=382226 [on 2015-09-23]

[B24] Gosliner TM Behrens DW Valdés A (2008) Indo-Pacific nudibranchs and sea slugs. A field guide to the World’s most diverse fauna. Sea Challengers Natural History Books, Etc., Washington, USA/ California Academy of Sciences, San Francisco, 426 pp.

[B25] Gouy M Guindon S Gascuel O (2010) SeaView version 4 : a multiplatform graphical user interface for sequence alignment and phylogenetic tree building. Molecular Biology and Evolution 27: 221–224. doi: 10.1093/molbev/msp259 19854763 10.1093/molbev/msp259

[B26] Grande C Templado J Cervera JL Zardoya R (2004a) Molecular phylogeny of Euthyneura (Mollusca: Gastropoda). Molecular Biology and Evolution 21: 303–313. doi: 10.1093/molbev/msh016 14660702 10.1093/molbev/msh016

[B27] Grande C Templado J Cervera JL Zardoya R (2004b) Phylogenetic relationships among Opisthobranchia (Mollusca: Gastropoda) based on mitochondrial *cox1*, *trnV*, and *rrnL* genes. Molecular Phylogenetics and Evolution 33: 378–388. doi: 10.1016/j.ympev.2004.06.008 15336672 10.1016/j.ympev.2004.06.008

[B28] Gray JE (1853) Revision of the families of nudibranch molluscs, with the description of a new genus of Phyllidiadae. Annals and Magazine of Natural History 1(2): 218–221. doi: 10.1080/03745485609496246

[B29] Guindon S Dufayard JF Lefort V Anisimova M Hordijk W Gascuel O (2010) New algorithms and methods to estimate maximum likelihood phylogenies: assessing the performance of PhyML 3.0. Systematic Biology 59: 307–321. doi: 10.1093/sysbio/syq010 20525638 10.1093/sysbio/syq010

[B30] Hoeksema BW (2007) Delineation of the Indo-Malayan Centre of maximum marine biodiversity: the Coral Triangle. In: Renema W (Ed.) Biogeography, Time and Place: Distributions, Barriers and Islands. Springer, Dordrecht, 117–178. doi: 10.1007/978-1-4020-6374-9_5

[B31] Hoeksema BW van der Meij SET (2008) Cryptic marine biota of the Raja Ampat Islands group. Naturalis, Leiden, 74 pp.

[B32] Hoeksema BW van der Meij SET (2010) Crossing marine lines at Ternate: Capacity building of junior scientist in Indonesia for marine biodiversity assessments. Naturalis, Leiden, 85 pp.

[B33] Lamarck JB (1801) Système des animaux sans vertèbres. Deterville, Paris, 64–66.

[B34] Lin G (1983) A study on the genus *Phyllidia* (Opisthobranchia) in China. Tropic Oceanology 2: 148–153.

[B35] Maeda T Kajita T Maruyama T Hirano Y (2010) Molecular phylogeny of the Sacoglossa, with a discussion of gain and loss of kleptoplasty in the evolution of the group. Biological Bulletin 219: 17–26. doi: 10.2307/27898985 20813986 10.1086/BBLv219n1p17

[B36] Mehrotra R Scott CM Rohrer JM Hoeksema BW (2015) Predation on a sacoglossan gastropod by a mushroom coral. Coral Reefs 34: 517. doi: 10.1007/s00338-015-1285-z

[B37] Milne-Edwards H (1848) Note sur la classification naturelle des mollusques gastéropodes. Annales des Sciences Naturelles, Zoologie, Ser. 3, 9: 102–112.

[B38] Penn O Privman E Ashkenazy H Landan G Graur D Pupko T (2010) GUIDANCE: a web server for assessing alignment confidence scores. Nucleic Acids Research 38: 23–28. doi: 10.1093/nar/gkq443 10.1093/nar/gkq443 PMC2896199 20497997

[B39] Piel WH (1991) Pycnogonid predation on nudibranchs and ceratal autotomy. Veliger 34: 366–367.

[B40] Pola M Gosliner TM (2010) The first molecular phylogeny of cladobranchian opisthobranchs (Mollusca, Gastropoda, Nudibranchia). Molecular Phylogenetics and Evolution 56: 931–941. doi: 10.1016/j.ympev.2010.05.003 20460158 10.1016/j.ympev.2010.05.003

[B41] Posada D (2008) jModelTest: Phylogenetic Model Averaging. Molecular Biology and Evolution 25: 1253–1256. doi: 10.1093/molbev/msn03 18397919 10.1093/molbev/msn083

[B42] Pruvot-Fol A (1957) Révision de la famille des Phyllidiadae (1). Journal de Conchyliologie, Paris 96: 55–80.

[B43] Puillandre N Lambert A Brouillet S Achaz G (2012) ABGD, Automatic Barcode Gap Discovery for primary species delimitation. Molecular Ecology 21: 1864–1877. doi: 10.1111/j.1365-294X.2011.05239.x 21883587 10.1111/j.1365-294X.2011.05239.x

[B44] Rafinesque C (1814) Précis des découvertes et travaux somiologiques de Mr. C.S. Rafinesque-Schmalz entre 1800 et 1814, ou choix raisonné de ses principals découvertes en Zoologiques et en Botaniques, pour servir d’introduction à ses ouvrages futurs. Published privately, Palerme, 55 pp.

[B45] Rambaut A (2009) FigTree 1.3.1. http://tree.bio.ed.ac.uk/software/figtree/

[B46] Ritson-Williams R Paul VJ (2007) Marine benthic invertebrates use multimodel cues for defense against reef fish. Marine Ecology Progress Series 340: 29–39. doi: 10.3354/meps340029

[B47] Ronquist F Huelsenbeck JP (2003) MRBAYES 3: Bayesian phylogenetic inference under mixed models. Bioinformatics 19: 1572–1574. doi: 10.1093/bioinformatics/btg180 12912839 10.1093/bioinformatics/btg180

[B48] Rudman WB Willan RC (1998) Opisthobranchia. In: Beesley PL Ross GJB Wells A (Eds) Mollusca: The Southern Synthesis. Fauna of Australia. CSIRO, Melbourne, 915–1035.

[B49] Sachidhanandam U Willan RC Chou LM (2000) Checklist of the nudibranchs (Opisthobranchia: Nudibranchia) of the South China Sea. The Raffles Bulletin of Zoology. Supplement 7: 513–537.

[B50] Schrödl M Jörger K Klussmann-Kolb A Wilson NG (2011) Bye bye “Opisthobranchia”! A review on the contribution of mesopsammic sea slugs to euthyneuran systematics. Thalassas 27: 101–112.

[B51] Shields CC Marko PB Woods HA Moran AL (2009) Nudibranchia in the Ross Sea, Antarctica: Lineage diversity and divergence estimated using methods of molecular phylogenetics and sequence divergence. Genbank, unpublished.

[B52] Tamura K Stecher G Peterson D Filipski A Kumar S (2013) MEGA6: Molecular Evolutionary Genetics Analysis version 6.0. Molecular Biology and Evolution 30: 2725–2729. doi: 10.1093/molbev/mst197 24132122 10.1093/molbev/mst197 PMC3840312

[B53] Thollesson M (2000) Increasing fidelity in parsimony analysis of dorid nudibranchs by differential weighting, or a tale of two genes. Molecular Phylogenetics and Evolution 16: 161–172. doi: 10.1006/mpev.2000.0789 10942604 10.1006/mpev.2000.0789

[B54] Turner LM Wilson NG (2008) Polyphyly across oceans: a molecular phylogeny of the Chromodorididae (Mollusca, Nudibranchia). Zoologica Scripta 37: 23–42. doi: 10.1111/j.1463-6409.2007.00310.x

[B55] Uribe RA Nakamura K Indacochea A Pacheco AS Hooker Y Schrödl M (2013) A review on the diversity and distribution of opisthobranch gastropods from Peru, with the addition of three new records. Spixiana 36: 43–60.

[B56] Valdés A (2001) Depth-related adaptations, speciation processes and evolution of colour in the genus *Phyllidiopsis* (Mollusca: Nudibranchia). Marine Biology 139: 485–496. doi: 10.1007/s002270100596

[B57] Valdés A (2002) A phylogenetic analysis and systematic revision of the cryptobranch dorids (Mollusca, Nudibranchia, Anthobranchia). Zoological Journal of the Linnean Society 136: 535–636. doi: 10.1046/j.1096-3642.2002.00039.x

[B58] Valdés A (2003) Preliminary molecular phylogeny of the radula-less dorids (Gastropoda: Opisthobranchia), based on 16S mtDNA sequence data. Journal of Molluscan Studies 69: 75–80. doi: 10.1093/mollus/69.1.75

[B59] Valdés A Gosliner TM (1999) Phylogeny of the radula-less dorids (Mollusca, Nudibranchia), with the description of a new genus and a new family. Zoologica Scripta 28: 315–360. doi: 10.1046/j.1463-6409.1999.00014.x

[B60] van Alphen J de Voogd NJ Hoeksema BW (2011) Differential feeding strategies in phyllidiid nudibranchs on coral reefs at Halmahera, northern Moluccas. Coral Reefs 30: 59. doi: 10.1007/s00338-010-0698-y

[B61] van der Meij SET Reijnen BT (2012) First observations of attempted nudibranch predation by sea anemones. Marine Biodiversity 42: 281–283. doi: 10.1007/s12526-011-0097-9

[B62] van Hasselt JC (1824) Uittreksel uit eenen brief van Dr J.C. van Hasselt, aan Prof. van Swinderen. Algemeene Konst- en Letter-bode voor het jaar 1824, 2: 20–24.

[B63] Vonnemann V Schrödl M Klussmann-Kolb A Wägele H (2005) Reconstruction of the phylogeny of the Opisthobranchia (Mollusca: Gastropoda) by means of 18S and 28S rRNA gene sequences. Journal of Molluscan Studies 71: 113–125. doi: 10.1093/mollus/eyi014

[B64] Wägele H Klussmann-Kolb A Verbeek E Schrödl M (2014) Flashback and foreshadowing – a review of the taxon Opisthobranchia. Organisms, Diversity & Evolution 14: 133–149. doi: 10.1007/s13127-013-0151-5

[B65] Wollscheid E Wägele H (1999) Initial results on the molecular phylogeny of the Nudibranchia (Gastropoda, Opisthobranchia) based on 18S rDNA data. Molecular Phylogenetics and Evolution 13: 215–226. doi: 10.1006/mpev.1999.0664 10603252 10.1006/mpev.1999.0664

[B66] Wollscheid-Lengeling E Boore J Brown W Wägele H (2001) The phylogeny of Nudibranchia (Opisthobranchia, Gastropoda, Mollusca) reconstructed by three molecular markers. Organisms Diversity & Evolution 1: 241–256. doi: 10.1078/1439-6092-00022

[B67] Yonow N (1986) Red Sea Phyllidiidae (Mollusca, Nudibranchia) with descriptions of new species. Journal of Natural History 20: 1401–1428. doi: 10.1080/00222938600770941

[B68] Yonow N (1996) Systematic revision of the family Phyllidiidae in the Indian Ocean Province: Part 1 (Opisthobranchia: Nudibranchia: Doridoidea). Journal of Conchology 35: 483–516.

[B69] Yonow N (2008) Sea slugs of the Red Sea. Pensoft, Sofia–Moscow, 304 pp.

[B70] Yonow N (2011) Results of the Rumphius Biohistorical Expedition to Ambon (1990). Part 15. The suborder Doridina (Mollusca, Gastropoda, Opisthobranchia, Nudibranchia). Zoologische Mededelingen, Leiden 85: 905–956.

[B71] Yonow N (2015) Sea slugs: unexpected biodiversity and distribution. In: Rasul NMA Stewart ICF (Eds) The Red Sea. Springer, Berlin, 531–550. doi: 10.1007/978-3-662-45201-1_30

[B72] Yonow N Anderson RC Buttress SG (2002) Opisthobranch molluscs from the Chagos Archipelago, Central Indian Ocean. Journal of Natural History 36: 831–882. doi: 10.1080/00222930110039161

