# Supplementary material for: Phylogenetic relationships within the Phyllidiidae (Opisthobranchia, Nudibranchia)
Source: Zookeys. 2016 Jul 14;(605):1–35. doi: 10.3897/zookeys.605.7136 (PMC4978068; doi:10.3897/zookeys.605.7136)
Supplement: Supplementary material 1 — COI sequences of lost Phyllidiidae specimens [file zookeys-605-001-s001.pdf]

Appendix. COI sequences of Phyllidiidae specimens that dried out after sequencing (numbers and localities are indicated in Table 1.

058 *Phyllidia elegans*

GGTCTTTTGGGAACAGGTTTAAGATTACTAATTCGTTTTGAGTTAGGGACTGCTGGGCCT
TTTTTAGGAGATGACCATTTTTATAATGTTATTGTAACAGCTCATGCTTTTGTAATAATT
TTTTTTCTAGTTATGCCTTTAATAATTGGAGGATTTGGAAACTGGATGGTTCCTTTACTA
ATTGGAGCTCCTGATATAAGTTTTCCTCGAATAAATAATATAAGGTTTTGATTACTTCCA
CCATCTTTTATTTTATTGTTGTGTTCAACTTTAATAGAAGGTGGTGCCGGAACAGGATGG
ACAGTTTACCCTCCTTTATCTGGTCCAATAAGACATGGTGGAACATCTGTCGATTTAGTA
ATTTTTTCTTTACACTTAGCTGGAGCCTCTTCTCTTCTTGGAGCAATTAATTTTATTACT
ACTATTTTTAATATGCGTTCACCTGCTATAACAATAGAACGTCTAAGGTTATTCGTTTGG
TCTGTTTTGGTAACAGCTTTTCTTTTACTTCTTTCTCTCCCTGTTCTGGCAGGAGCTATT
ACTATACTTTTGACAGATCGAAATTTTAATACAAGCTTTTTTGATCCAGCTGGTGGAGGA
GAC

137 *Phyllidia elegans*

GGTCTTTTGGGAACAGGTTTAAGATTACTAATTCGTTTTGAGTTAGGGACTGCTGGGCCT
TTTTTAGGAGATGACCATTTTTATAATGTTATTGTAACAGCTCATGCTTTTGTAATAATT
TTTTTTCTAGTTATGCCTTTAATAATTGGAGGATTTGGAAACTGGATGGTTCCTTTACTA
ATTGGAGCTCCTGATATAAGTTTTCCTCGAATAAATAATATAAGGTTTTGATTACTTCCA
CCATCTTTTATTTTATTGTTGTGTTCAACTTTAATAGAAGGTGGTGCCGGAACAGGATGG
ACAGTTTACCCTCCTTTATCTGGTCCAATAGGACATGGTGGAACATCTGTCGATTTAGTA
ATTTTTTCTTTACATTTAGCTGGAGCCTCTTCTCTTCTTGGAGCAATTAATTTTATTACT
ACTATTTTTAATATGCGTTCACCTGCTATAACAATAGAACGTCTAAGGTTATTCGTTTGG
TCTGTTTTGGTAACAGCTTTTCTTTTGCTTCTTTCTCTCCCTGTTCTGGCAGGAGCTATT
ACTATACTTTTGACAGATCGAAATTTTAATACAAGCTTTTTTGATCCAGCTGGTGGAGGA
GAC

156 *Phyllidia elegans*

GGTCTTTTGGGAACAGGTTTAAGATTACTAATTCGTTTTGAGTTAGGGACTGCTGGGCCT
TTTTTAGGAGATGACCATTTTTATAATGTTATTGTAACAGCTCATGCTTTTGTAATAATT
TTTTTTCTAGTTATGCCTTTAATAATTGGAGGATTTGGAAACTGGATGGTTCCTTTACTA
ATTGGAGCTCCTGATATAAGTTTTCCTCGAATAAATAATATAAGGTTTTGATTACTTCCA
CCATCTTTTATTTTATTGTTGTGTTCAACTTTAATAGAAGGTGGTGCCGGAACAGGATGG
ACAGTTTACCCTCCTTTATCTGGTCCAATAGGACATGGTGGAACATCTGTCGATTTAGTA
ATTTTTTCTTTACATTTAGCTGGAGCCTCTTCTCTTCTTGGAGCAATTAATTTTATTACT
ACTATTTTTAATATGCGTTCACCTGCTATAACAATAGAACGTCTAAGGTTATTCGTTTGG
TCTGTTTTGGTAACAGCTTTTCTTTTGCTTCTTTCTCTCCCTGTTCTGGCAGGAGCTATT
ACTATACTTTTGACAGATCGAAATTTTAATACAAGCTTTTTTGATCCAGCTGGTGGAGGA
GAC

074 *Phyllidia varicosa*

GGTCTTTTAGGAACAGGGTTAAGTTTGTTAATTCGTTTTGAATTAGGAACTGCTGGACCT
TTTCTAGGAGATGACCATTTTTATAATGTGATTGTAACGGCTCATGCTTTTGTAATAATT
TTCTTTTTAGTTATACCTTTGATAATTGGGGGATTTGGTAACTGAATAGTCCCTTTATTA
ATTGGAGCTCCAGATATAAGTTTCCCCCGAATAAATAATATAAGTTTTTGGTTACTTCCA
CCCTCTTTTATTTTATTACTATGTTCGACCTTGATAGAAGGTGGAGCCGGAACAGGATGG
ACTGTTTACCCTCCATTATCCGGTTCAATAGGTCATGGGGGAACATCTGTTGATTTAGTA
ATTTTTTCTTTACACTTAGCAGGGGCTTCTTCTTTATTAGGAGCTATTAATTTCATTACT
ACTATTTTTAATATACGTTCACCTGCTATAACAATGGAACGTTTAAGTTTATTTGTTTGA
TCAGTTCTGGTGACTGCTTTCCTTTTACTTCTTTCCTTACCAGTTTTGGCAGGAGCTATT
ACTATACTTTTGACAGATCGTAATTTTAATACTAGATTTTTTGACCCAGCAGGTGGAGGA
GAT

75F *Phyllidiella pustulosa*

GGTTTATTAGGAACGGGATTAAGATTATTAATTCGTTTTGAGTTAGGTGCCGCTGGTGCT
TTTTTGGGGGATGACCACTTTTATAATGTAATTGTGACTGCCCATGCTTTCGTTATAATT
TTTTTTTTGGTAATGCCTTTAATAATTGGAGGATTTGGAAATTGAATGGTACCTTTGTTA
ATTGGGGCTCCARACATGAAATTTCCTCGAATAAATAATATAARATTTTGGTTGTTGCCC
CCTTCGTTTATTCTTCTTTTATGCTCAACATTGATAGAGGGAGGGGCGGGGACAGGTTGA
ACTGTCTATCCTCCTTTATCTGGGGCAATAGGGCACGGAGGAACTTCAGTTGATCTTGCT
ATTTTTTCTCTTCATTTAGCTGGAGCTTCTTCTTTACTGGGAGCAATTAATTTTATTACT
ACTATTTTTAATATGCGATCTCCTGCGATGACAATARAACGATTAACCCTATTTGTTTGA
TCTGTTCTAGTAACCGCTTTCTTGTTGCTTTTATCTCTTCCAGTATTAGCTGGGGCTATT
ACTATGCTATTAACARATCGRAATTTTAATACGAGATTTTTTGACCCA
